# Disrupting biological sensors of force promotes tissue regeneration in large organisms

**DOI:** 10.1038/s41467-021-25410-z

**Published:** 2021-09-06

**Authors:** Kellen Chen, Sun Hyung Kwon, Dominic Henn, Britta A. Kuehlmann, Ruth Tevlin, Clark A. Bonham, Michelle Griffin, Artem A. Trotsyuk, Mimi R. Borrelli, Chikage Noishiki, Jagannath Padmanabhan, Janos A. Barrera, Zeshaan N. Maan, Teruyuki Dohi, Chyna J. Mays, Autumn H. Greco, Dharshan Sivaraj, John Q. Lin, Tobias Fehlmann, Alana M. Mermin-Bunnell, Smiti Mittal, Michael S. Hu, Alsu I. Zamaleeva, Andreas Keller, Jayakumar Rajadas, Michael T. Longaker, Michael Januszyk, Geoffrey C. Gurtner

**Affiliations:** 1 Department of Surgery, Division of Plastic and Reconstructive Surgery, Stanford University School of Medicine, Stanford, CA USA; 2 University Center for Plastic, Reconstructive, Aesthetic and Hand Surgery, University Hospital Regensburg and Caritas Hospital St. Josef, Franz-Josef-Strauss-Allee 11, Regensburg, Germany; 3 Clinical Bioinformatics, Saarland University, Saarbruecken, Germany; 4 Biomaterials and Advanced Drug Delivery Laboratory, Stanford University, Stanford, CA USA; 5 Department of Neurology & Neurological Sciences, Stanford University, Stanford, CA USA

**Keywords:** Regenerative medicine, Mechanotransduction, Mechanisms of disease, Regeneration, Experimental models of disease

## Abstract

Tissue repair and healing remain among the most complicated processes that occur during postnatal life. Humans and other large organisms heal by forming fibrotic scar tissue with diminished function, while smaller organisms respond with scarless tissue regeneration and functional restoration. Well-established scaling principles reveal that organism size exponentially correlates with peak tissue forces during movement, and evolutionary responses have compensated by strengthening organ-level mechanical properties. How these adaptations may affect tissue injury has not been previously examined in large animals and humans. Here, we show that blocking mechanotransduction signaling through the focal adhesion kinase pathway in large animals significantly accelerates wound healing and enhances regeneration of skin with secondary structures such as hair follicles. In human cells, we demonstrate that mechanical forces shift fibroblasts toward pro-fibrotic phenotypes driven by ERK-YAP activation, leading to myofibroblast differentiation and excessive collagen production. Disruption of mechanical signaling specifically abrogates these responses and instead promotes regenerative fibroblast clusters characterized by AKT-EGR1.

## Abstract (web summary)

Humans and other large mammals heal wounds by forming fibrotic scar tissue with diminished function. Here, the authors show that disrupting mechanotransduction through the focal adhesion kinase pathway in large animals accelerates healing, prevents fibrosis, and enhances skin regeneration.

## Introduction

Fibrosis is a significant cause of morbidity and mortality and contributes to 45% of all deaths in the United States^1^. In humans and other large organisms, injured tissue heals by fibrosis and scar formation, which significantly impacts organ function, as seen in myocardial infarction and ischemic stroke^2^. In contrast, smaller model organisms, such as planaria and salamanders^3,4^, heal by regenerating normal tissue architecture without fibrosis^5^. Achieving scarless tissue regeneration in humans and other large organisms remains the holy grail of biomedical research with the potential to revolutionize patient care for many fibrotic diseases^6^.

A key feature that distinguishes model organisms from humans and other large mammals is body mass^7^. Scaling principles dictate that as organism size increases, the peak stresses transduced to tissues during movement increases exponentially^7–9^. Large organisms have evolved to compensate for these increased forces through a variety of mechanisms; from fundamental changes (e.g., tissue hypertrophy) to complex adaptations (e.g., alterations in posture to reduce forces experienced by bone and muscle during locomotion)^9,10^. Skin in particular has adapted to elevated mechanical stress by promoting a hypertrophic healing response, resulting in the formation of dysfunctional scar tissue^9,10^.

Efficient approaches to prevent the development of skin fibrosis in humans have been limited, and there are currently no therapeutics that promote wound healing or reverse fibrosis. The vast majority of in vivo studies in wound healing are conducted in rodents^11–13^. However, rodents are several magnitudes smaller in mass than humans and experience lower tissue forces^7^, and even though current wound models try to mimic human-like wound biology, these models still do not fully replicate human scar formation and fibrosis^12,14^. These fundamental differences have significantly limited the translational relevance of fibrosis studies performed in rodent models. To effectively translate therapies for human clinical use, we must thoroughly investigate potential therapies in both clinically relevant, large animal models as well as in human cells.

Focal adhesion kinase (FAK) is a well-characterized transducer of tissue-level integrin-matrix forces to downstream intracellular pathways^13^. Pharmacologic inhibition of FAK has been demonstrated to be a safe approach for the treatment of advanced solid tumors in clinical trials^15^. We have previously shown that disruption of FAK reduces inflammation and fibrosis in rodent models^12,16^. To evaluate the effects of blocking mechanotransduction in tissue repair in large mammals, we created deep partial-thickness excisional wounds on the dorsum of red Duroc pigs, the closest large animal model to human wound healing^17^. Partial-thickness injuries, which could be caused by trauma, oncologic resection, venous stasis, or burns, are the most common wounds treated in clinical practice^18^. These wounds are often left to heal by secondary intention and frequently result in debilitating scar formation and contracture^19^. Both humans and red Duroc pigs heal from deep dermal injuries by developing collagenous hypertrophic scars (HTS) that replace the physiologic skin tissue^14,20^. The resulting scar tissue is characterized by a thicker dermis, increased mechanical stiffness, and an absence of both skin appendages (e.g., hair follicles) and intradermal adipose tissue^21^. Here, we show that blocking mechanical signaling via FAK inhibition promotes regenerative healing, defined by formation of healed skin with (1) restored biomechanical properties, (2) hair follicle regrowth, and (3) normal collagen fiber architecture. To confirm these findings in humans, we quantify the effects of FAK inhibition on three-dimensional (3D) cultured human fibroblasts at the cellular and transcriptomic levels using single-cell RNA sequencing. We demonstrate that mechanical stress induces profibrotic fibroblast differentiation fates in large organisms, which can effectively be averted by FAK inhibition to instead induce discrete fibroblast clusters that promote wound regeneration.

## Results

### Inhibiting FAK in large mammals allows tissue regeneration

To evaluate the effects of blocking mechanotransduction on tissue repair in large animals, we employed a pharmacologic inhibitor of FAK (FAKI) (VS-6062) in red duroc pigs. VS-6062 (formerly known as PF-562,271) has been well characterized as having high specificity for FAK over a wide panel of other kinases^22,23^. We first measured local and systemic toxicity and observed that neither topical administration of FAKI solution nor subcutaneous implantation of FAKI hydrogels resulted in any adverse reactions in unwounded porcine skin (Supplementary Fig. 1a, b). Furthermore, porcine serum FAKI concentrations following local treatment were almost undetectable and less than 1% of the maximum tolerated human dose observed in a previous Phase 1 clinical trial (Supplementary Fig. 1c)^24^.

**Fig. 1:**
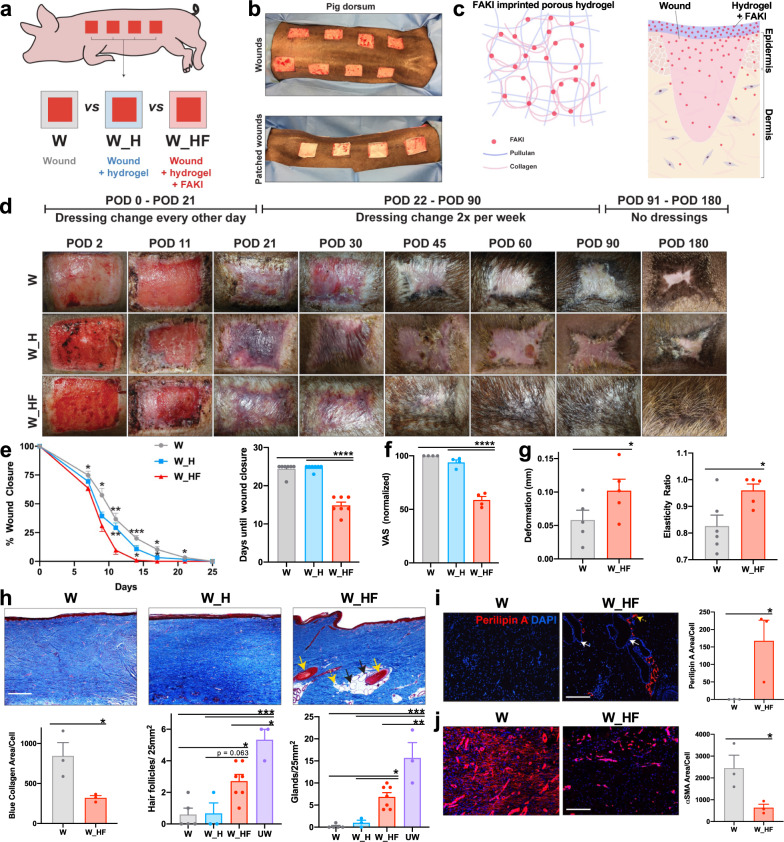
Disruption of mechanotransduction in large organisms accelerates deep partial-thickness wound healing, attenuates fibrotic scar formation, and promotes tissue regeneration. **a, b** Large area (25 cm^2^) deep partial-thickness excisional wounds were created on the lateral dorsum (left and right) of red Duroc pigs. Wounds were either treated with standard bandage dressings (Wounded: W, gray), blank pullulan-collagen hydrogels (Wound + Hydrogel: W_H, blue), or FAKI-releasing hydrogels (Wound + FAKI hydrogel: W_HF, red). All wounds were evaluated by gross photography at indicated timepoints until postoperative day (POD) 180. Dressing changes and hydrogel treatments in all pigs continued until POD90. **c** Schematic of hydrogel delivery of FAKI to the wound. **d** Representative images tracking wound closure and scar formation over time. **e** Wound closure rates (*p < 0.05*;* **p < 0.01*;* ***p < 0.001*;* ****p = 0.0001; *n* = 7 independent wounds per condition) and **f** Visual Analog Scale (VAS*)* scoring (****p *=* *0.0001)* were assessed by four blinded scar experts from digital photographs of wounds throughout the healing process (wound closure assessed from POD 0 to POD 25; VAS assessed at POD90) (*n* = 4 independent blinded scores of 7 wounds per condition). **g** Wound firmness (left, *p = 0.0357) and elasticity (right, *p = 0.023) were compared between W and W_HF by cutometer at POD 60 (*n* = 8 independent wounds per condition). **h** Masson’s Trichrome staining of healed scar to assess the presence of hair follicles (yellow solid arrows), secondary cutaneous glands (black solid arrows), and intradermal adipocytes proximal to the appendage structures (yellow dashed arrows). Scale bar: 200 µm. Blinded experts counted the hair follicles (***p = 0.0005*,* *p = 0.021) and cutaneous glands (***p = 0.0001*,* **p = 0.0026*,* *p = 0.0445). Collagen blue area quantified with custom MATLAB algorithm (*p = 0.0361). **i** Perilipin staining and quantification (*n* = 3 independent wounds, *p = 0.0472). Scale bar: 200 µm. **j** αSMA staining and quantification (*n* = 3 independent wounds, *p = 0.0408). Scale bar: 200 µm. Statistical comparisons were made either by using either a one-way (**f, h**) or two-way (**e**) analysis of variance (ANOVA) with Tukey’s multiple comparisons tests when comparing more than two groups or using paired (**g**) or unpaired (**i, j**) two-tailed *t*-tests when comparing two groups. Each datapoint represents an independent wound. All data represent mean ± SEM. Representative images are shown from similar images across all wounds.

FAKI was delivered to partial-thickness excisional wounds on the dorsum of red Duroc pigs in a controlled manner, using a biodegradable and biocompatible pullulan-collagen-based hydrogel scaffold (Fig. 1a-c; Supplementary Fig. 1d)^11^. We found that wounds treated with FAKI hydrogels (W_HF) had fully healed at postoperative day (POD) 14 ± 2.3, more than 10 days earlier than wounds treated with standard dressings (W) or empty hydrogels (W_H) (****p < 0.0001) (Fig. 1d, e). Importantly, this pharmacologic blockade of mechanical signaling resulted in skin that had less scar formation with more hair upon gross inspection, at PODs 40, 60, and 90 (Fig. 1f; Supplementary Fig. 1e). Using a tissue cutometer, we demonstrated that FAKI-treated wounds were less firm and more elastic than untreated wounds and exhibited more similar biomechanical properties to unwounded skin (Fig. 1g).

Wound tissue treated with FAKI exhibited dramatic regrowth of hair follicles and subcutaneous glands (sweat and sebaceous) (Fig. 1h), as well as newly regenerated peri-follicular adipose tissue, demonstrated by immunofluorescent staining for the adipocyte marker perilipin A (Fig. 1i). In contrast, control wounds failed to regenerate secondary structures and instead exhibited increased collagen deposition, fibrosis (Fig. 1h), and an increased number of alpha smooth muscle actin (αSMA) expressing myofibroblasts (Fig. 1j), a key cell that is known to drive tissue fibrosis and scar formation^1,2^.

**Fig. 2:**
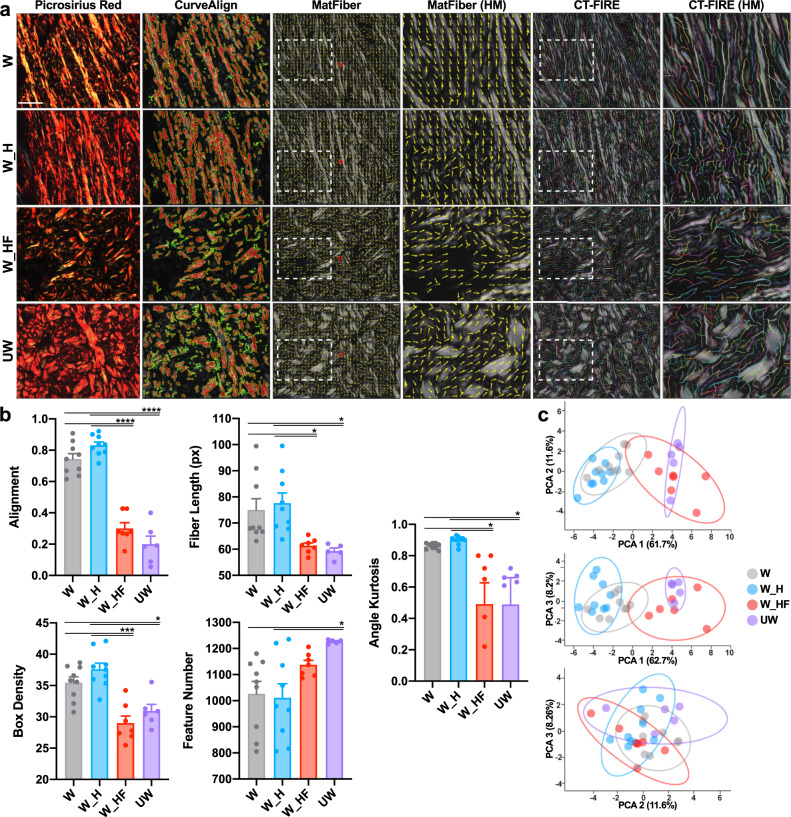
FAKI-mediated inhibition of mechanotransduction in wounds of large organisms promotes a regenerative organization of collagen fiber networks. **a** Picrosirius Red staining of postoperative day 90 standard wounds (W, gray, *n* = 9 independent wounds), blank pullulan-collagen hydrogels treated wounds (W_H, blue, *n* = 9 independent wounds), or FAKI-releasing hydrogel treated wounds (W_HF, red, *n* = 7 independent wounds) was quantified and compared to unwounded skin (UW, purple, *n* = 6 independent skin samples) using alignment (CurveAlign, second column), fiber length metrics (MatFiber, middle two columns), and CT-Fire (right two columns). Scale bar: 10 µm. **b** Quantification of the different collagen fiber network characteristics, alignment (****p = 0.0001), fiber length (*p = 0.0494), box density (***p = 0.0006*;* *p = 0.0275), feature number (*p = 0.0196), and angle kurtosis (*p = 0.0394) across the four different groups. **c** Principal component analysis (PCA) plots showing the variance explained by the first three principal components (PCs); PC1 explains 61.7% of the variance, PC2 explains 11.6%, and PC3 explains 8.2%. Statistical comparisons were made using a one-way analysis of variance (ANOVA) with Tukey’s multiple comparisons tests. Each datapoint represents an independent wound. All data represent mean ± SEM. Representative images are shown from similar images across all wounds.

We performed a detailed quantitative assessment of the collagen architecture of the wounds at POD90, using the software algorithms CT-FIRE, CurveAlign, and MatFiber, which have all been previously developed to analyze collagen fiber properties in histologic images^25–27^. Control wounds were found to exhibit a significant disruption of dermal architecture across these metrics, with collagen fiber elongation and increased unidirectional alignment^28^ (Fig. 2a). Wounds with pharmacological blockade of FAK, by contrast, healed with a basket weave-like collagen fiber network similar to unwounded skin across a wide range of metrics (Fig. 2a). Specifically, FAK-inhibited wounds and unwounded skin both demonstrated decreased alignment, fiber length, angle kurtosis, and box density, while also both having an increased number of shorter collagen fibers (*p < 0.05) (Fig. 2b). Utilizing all 24 collagen structural parameters from these analyses (Supplementary Fig. 2), we performed a principal component analysis (PCA) and found that the first principal component (PC1) distinctly separated the fibrotic wounds (W, W_H) from regenerative healing (W_HF, UW) (Fig. 2c). We can interpret PC1 as an axis of fibrosis-regeneration that quantifies the significant collagen structural differences between fibrosis and regeneration across these 24 parameters.

### Manipulating mechanical forces modulates fibrotic behavior

**Fig. 3:**
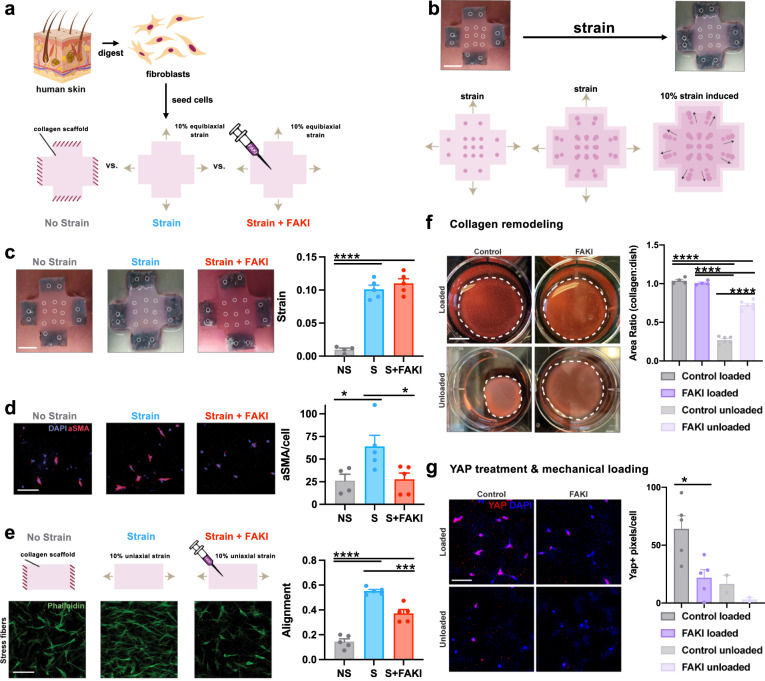
3D collagen scaffold system recapitulates observations seen in the porcine tissue. **a** Freshly isolated fibroblasts were seeded into 3D collagen scaffolds and either restrained (no strain, NS, gray) or subjected to strain (S, blue) or strain + 10 μM FAKI (S + FAKI, red). **b, c** We used titanium oxide dots (inner 9 circles) to track and quantify the exact imposed strains on the collagen scaffolds, shown by gross photography (top row) and a schematic (bottom row). Scaffolds were pinned at the arms to enforce the strain (outer circles; 2 circles per arm) (****p = 0.0001). Scale bar: 1 cm. **d** Alpha smooth muscle actin (αSMA) myofibroblast protein expression in fibroblasts cultured in all three conditions was quantified by immunofluorescence staining. (NS: *n* = 4 independent collagen scaffolds; S and S + FAKI: *n* = 5 independent collagen scaffolds per group; *p < 0.0462). DAPI = blue, αSMA = red. Scale bar: 140 µm. **e** Alignment of fibroblasts within 3D stretch culture system that underwent uniaxial restraint (no strain), 10% uniaxial strain only, or 10% uniaxial strain + FAKI was quantified using a previously published algorithm to analyze fibroblasts immunostained for phalloidin (green, *n* = 5; ****p = 0.0001*,* ***p = 0.0005)^26^. Scale bar: 140 µm. **f** Contraction in vitro assay using collagen scaffolds to quantify remodeling of the ECM environment (****p = 0.0001). Scale bar: 1 cm. **g** Pharmacological unloading (FAKI treatment) and mechanical unloading create similar decreases in YAP expression. Control (grey) & FAKI (purple) loaded (*n* = 5 independent collagen scaffolds; *p = 0.0258); control (light gray) & FAKI (light purple) unloaded (*n* = 2 independent collagen scaffolds). Scale bar: 140 µm. Statistical comparisons were made by using a one-way analysis of variance (ANOVA) with Tukey’s multiple comparisons tests (**c–g**). Each datapoint represents an independent collagen scaffold. All data represent mean ± SEM. Representative images are shown from similar images across all experiments.

To understand how disruption of mechanotransduction may be relevant to human healing, we investigated the behavior of human fibroblasts in response to changes in tissue force. We employed a three-dimensional (3D) culture system that permits the precise manipulation of mechanical strain (and therefore stress) applied to cells (Fig. 3a-c)^26^. Fibroblasts were isolated from human tissue samples, seeded within 3D collagen scaffolds, and either restrained (no strain, NS) or subjected to 10% strain (Strain, S) or 10% strain with FAK inhibition (Strain + FAKI, S + FAKI). We have previously demonstrated that fibroblasts cultured in this system display physiologic morphology and actin/stress fiber machinery matching fibroblasts in mechanically stressed in vivo scar environments^26,29^. To further demonstrate that our culture system accurately recreates the in vivo wound environment, we also observed increases in αSMA+ myofibroblasts induced by mechanical strain (Fig. 3d), matching observations seen in vivo (Fig. 1j). When cultured in a uniaxial strain environment^26^, these fibroblasts demonstrated elongated, unidirectional cellular alignment similar to the highly aligned fibrotic scar observed in human and porcine scars^28,30^ (Fig. 3e), while fibroblasts blocked from sensing mechanical forces demonstrated a multi-directional organization similar to native skin architecture^28^ (Fig. 2a, b).

Fibroblasts play a critical role in reorganizing extracellular matrix (ECM) by depositing and remodeling collagen to develop long, aligned fibers^31^. Using a contraction assay, we observed that fibroblasts lost their ability to remodel the surrounding ECM environment upon FAKI treatment (Fig. 3f)^13^. This attenuation of remodeling was also correlated with a decrease in YAP expression, a downstream transcription factor of the FAK pathway (Fig. 3g)^32,33^. These data demonstrate that our 3D human culture system provides a physiological mechanical environment that induces fibroblast phenotypes consistent with previous findings on αSMA and YAP mechanotransduction expression during collagen remodeling and deposition^11,13,32^. During the process of wound healing, increased mechanical forces trigger activation of FAK and increase integrin-ECM connections, which in turn promote stabilization of the f-actin cytoskeleton, αSMA expression, and cellular tension^34^. αSMA stabilization promotes expression of transnuclear proteins, such as YAP, which in turn translocates into the nucleus to activate a cascade of profibrotic and mechanotransduction signaling^33^. Since our 3D culture system accurately recreates the in vivo mechanical environment by inducing fibrotic myofibroblast phenotypes, we then used the system to examine specific cellular signaling pathways governing regeneration or fibrosis in human fibroblasts.

### Mechanotransduction inhibition induces regenerative programs

We have previously shown that targeting mechanotransduction with FAKI in a murine model of hypertrophic scar (HTS) disrupts the FAK-ERK-MCP-1 pathway and reduces the expression of those specific fibrotic and inflammatory signals^13^. However, these previous small animal studies were limited in scope and unable to fully examine the plethora of cellular signaling pathways, which are altered in response to mechanical stress disruption. Recent advances in single-cell transcriptomics have increased our ability to explore heterogeneous cellular responses, such as those associated with modulation of mechanical forces^35^.

**Fig. 4:**
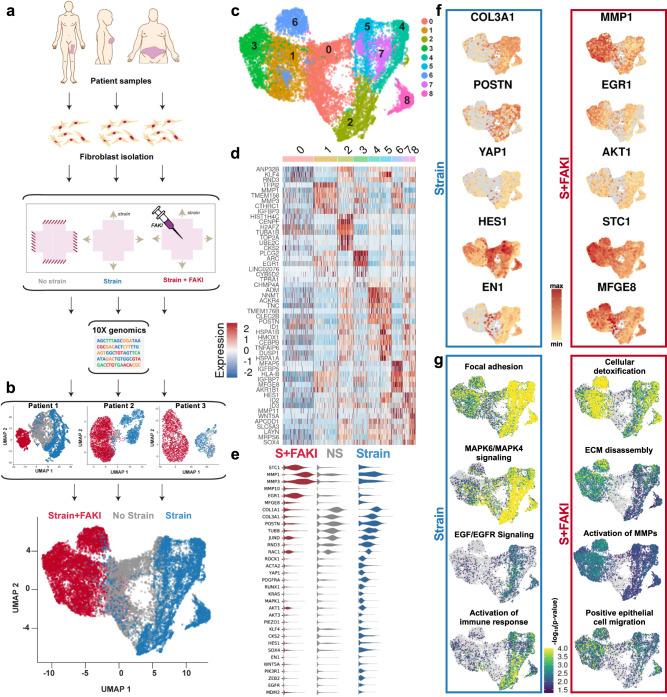
Mechanical stress drives profibrotic fibroblast heterogeneity; subsequent inhibition of mechanotransduction reduces heterogeneity and triggers AKT-dependent EGR1 and MFGE8 expression. **a** Adult human dermal fibroblasts were isolated from tissue collected from three patients at different anatomical locations: the breast skin from a mastectomy sample, the abdomen skin from an abdominoplasty sample, and the thigh skin from a thighplasty sample. Freshly isolated fibroblasts were seeded into 3D collagen scaffolds and subjected to either no strain (NS, gray), strain (S, blue), or strain +10 μM FAKI (S + FAKI, red) and then submitted for 10× genomics. **b** UMAP embeddings of cellular transcription profiles for the three patients were combined into a final embedding. **c** Unsupervised clustering of fibroblast transcriptional signatures revealed a total of 9 distinct clusters of human dermal fibroblasts (clusters 0–8). **d** Heatmap of the top five differentially expressed genes in all clusters. **e** Violin plots of group-defining differentially expressed genes. **f** Gene expression of group-defining genes projected onto UMAP embedding. **g** Over-representation analysis (ORA) of key pathways that differentiate the groups projected onto UMAP embedding.

To examine the molecular drivers of fibrosis and regeneration in human fibroblasts, we used single-cell RNA sequencing (scRNA-seq) in combination with our 3D collagen scaffold system (Fig. 4a). Fibroblasts were isolated from a variety of anatomic locations, and each patient’s fibroblasts were separately subjected to the previously mentioned NS, S, and S + FAKI conditions. After 48 h, the collagen scaffolds were enzymatically digested and scRNA-seq was performed on the cells using the 10× Genomics Chromium platform^35^. Data for individual cells were subjected to Louvain-based clustering and embedded into a two-dimensional UMAP space in a manner blinded to the phenotype of origin^36^. Overall, we found that mechanical strain shifted fibroblast transcriptional programs away from the NS cell states (Fig. 4b). Conversely, FAKI treatment pushed the cells to a new transcriptional metastate, and this shift was present even when analyzing each set of patient’s cells individually (Fig. 4b, top three plots). These findings highlight the robustness of FAKI treatment across three separate tissue donor sites collected on three different days.

UMAP-based clustering identified nine transcriptionally distinct fibroblast clusters (clusters 0–8) in the pooled dataset (Fig. 4c). Unstrained fibroblasts were found to aggregate together as a relatively homogeneous group, representing the majority of cells in our putative cluster 0. These cells, defined primarily by consistent expression of fibroblast housekeeping genes such as *RND3*, likely represent the native fibroblast steady-state in our system (Fig. 4c, d)^37^. By contrast, mechanical strain strongly altered transcriptomic profiles and considerably increased fibroblast transcriptional heterogeneity. Strained fibroblasts differentiated among five different heterogeneous clusters, delineated by clusters 2, 4, 5, 7, and 8 (Fig. 4b, c). Overall, all of these strained fibroblasts were defined by collagen production (*COL1A1, COL3A1)*, myofibroblast differentiation (*POSTN, ACTA2 [encoding αSMA], PDGFRA, RUNX1*, *ZEB2*)^34,38,39^, and genes related to mechanotransduction *PTK2* (encoding FAK) and its downstream effector *YAP1* (Fig. 4f, Supplementary Fig. 3a-c), recapitulating our findings identifying αSMA and YAP upregulation both in vivo (Fig. 1g) and in vitro (Fig. 3d, g). We also observed overall upregulation of *EN1* (encoding engrailed-1) and *PRRX1*, which our group has previously identified as hallmarks of a profibrotic fibroblast lineage in small animal models (Fig. 4f; Supplementary Fig. 3a-c)^40,41^.

Next, we used Genetrail3, a computational pipeline for over-representation analysis (ORA) of specific genesets on a single-cell level, to further investigate differential regulation of cellular signaling pathways among human fibroblast groups^42^. We found that mechanically strained fibroblasts exhibited a significant induction of genesets for focal adhesion (WP306), MAPK6/MAPK4 signaling (WP3307), positive regulation of actin filament binding assembly (GO-BP:0032233), TGF-beta signaling pathway (KEGG:M2642), and EGF/EGFR signaling pathway (WP437) (Fig. 4g; Supplementary Fig. 3d), with an enrichment of the genes *EGFR, MAPK1, ROCK1*, and *RAC1* (Supplementary Fig. 3a, c)^43–47^. Furthermore, strained fibroblasts showed a significant enrichment of activated immune response pathways (GO-BP:0002253) (Fig. 4g) and positive regulation of the immune response (GO-BO:0002218) (Supplementary Fig. 4b), corresponding to upregulation of inflammatory genes such as *CCL2* (Supplementary Fig. 3a). We have previously shown that MCP-1 (protein form of *CCL2*) is an inflammatory cytokine upregulated by fibroblasts in response to mechanical activation during murine fibrosis^13^. These findings further demonstrate that mechanical strain promotes inflammation by upregulating fibroblast MCP-1 expression, contributing to fibro-proliferation that attracts inflammatory cells to the wound site and further aggravates inflammation and fibrosis.

The strained fibroblast clusters also expressed considerable heterogeneity, representing several differentiation fates that each contribute to the development of fibrosis. Cluster 2 cells upregulated *CKS2*, which increases cellular proliferation and metabolic activity^48^, and demonstrated an enrichment for cell division pathways (GO-BP:0051301), signifying a highly proliferative state (Supplementary Fig. 4b). Especially mechanosensitive clusters 4, 5, and 7 demonstrated upregulation of pathways for the alteration of the YAP1/ECM axis (WP3967) (Supplementary Fig. 4b), corresponding with increased activation of myofibroblast differentiation gene *POSTN* (Fig. 4f). Clusters 5 and 8 represented especially fibrotic clusters, with an enrichment for fibrosis pathways (WP3624) (Supplementary Fig. 4b) and upregulation of *HES1* and *SOX4* (Fig. 4d-e; Supplementary Fig. 3a-c). *HES1* is a downstream effector of *NOTCH3* and has been related to a variety of human fibrotic diseases^49^, and *SOX4* expression has been tightly linked to various profibrotic factors, such as TGFB, Wnt, and NOTCH^50–52^. Clusters 2 and 5 also both demonstrated positive regulation of the immune response (GO-BP:0002218), indicating proinflammatory phenotypes. Within each cluster, individual cells also each exhibited heterogeneity when expressing key genes (Supplementary Fig. 4a). From this analysis, cluster 2 could represent an early proliferative and proinflammatory fibroblast state, clusters 4 and 7 could represent mechanoresponsive fibroblast states that drive differentiation into myofibroblasts, and clusters 5 and 8 could represent fibroblasts found in chronic, late-stage fibrotic conditions.

Suppression of mechanical signaling by FAKI in strained fibroblasts abrogated nearly all of these transcriptomic signatures, effectively blocking fibroblast differentiation into profibrotic and proinflammatory myofibroblast clusters. Instead, we found that disruption of FAK signaling shifted fibroblasts toward a more homogeneous metastate (Fig. 4b, c). We observed that blocking mechanical signaling strongly reduced the transcription of collagen encoding genes, such as *COL1A1* and *COL3A1* (Fig. 4e, f), and strongly induced the expression of the matrix-metalloproteinase (MMP) genes *MMP1, MMP3*, and *MMP10* (Fig. 4e, f, Supplementary Fig. 3a-c). MMPs reduce fibrosis across a wide range of disease models through collagen degradation^53–55^, and also promote cellular migration and re-epithelialization^53,56^. Finally, FAK inhibition induced the expression of the antifibrotic gene *STC1* (stanniocalcin-1), which has also been shown to promote wound healing and re-epithelialization^57,58^.

We observed a significant reduction of the MAPK-ERK mechanotransduction pathway but preservation of AKT after blocking mechanical signaling with FAK inhibition (Fig. 4e, f, Supplementary Fig. 3a-c), consistent with prior findings^11,13^. Differential expression analysis revealed that FAK-inhibited fibroblasts upregulated *EGR1* (encoding early growth response protein 1) and *MFGE8* (encoding milk fat globule-EGF factor 8 protein or lactadherin) expression, which are both mediated by AKT signaling. Recent studies have found that the AKT-EGR1 pathway has been specifically associated with regenerative phenotypes that regulate tumor suppression^59–61^, while *MFGE8* mitigates scar by tagging collagen molecules for phagocytosis^62^. *MFGE8* also promotes regeneration by positively regulating vascular endothelial growth factor (VEGF) expression and angiogenesis through AKT phosphorylation^63^. FAK inhibition consistently promoted these regenerative transcriptional profiles, even when analyzing each set of patient cells individually (Supplementary Fig. 5a, b). Furthermore, we found that even cells experiencing a basal level of physiologic strain responded to FAK inhibition (No Strain + FAKI group) with similar shifts in gene expression by decreasing profibrotic signaling and increasing regenerative transcription (Supplementary Fig. 3e).

Genetrail3 analysis of FAK-inhibited fibroblasts demonstrated a significant induction of genesets for ECM disassembly (GO-BP:0022617), activation of MMPs (WP2769), and collagen catabolic process (GO-BP:0030574), consistent with an antifibrotic phenotype (Fig. 4g). Moreover, FAK inhibition strongly induced beneficial transcriptional genesets for cellular detoxification (GO-BP:1990748), epithelial cell migration (GO-BP:0010634), cellular homeostasis (GO-BP:0019725), cell redox homeostasis (GO-BP:0045454), and collagen catabolic processes (GO-BP:0030574) (Fig. 4g; Supplementary Fig. 3d; Supplementary Fig. 4b). Cluster 3 fibroblasts showed a specific enrichment for pathways related to tissue development (GO-BP:0009888) and adipogenesis (WP236), demonstrating that this cluster of cells could potentially have the highest regenerative potential (Supplementary Fig. 4b). Based on these findings, we postulated that FAK inhibition promotes collagen degradation and reduces profibrotic fibroblast phenotypes by inhibiting a wide range of mechanotransduction pathways, such as MAPK-ERK and YAP, while preserving the AKT pathway to induce regenerative phenotypes through EGR1 and MFGE8.

### Mechano-modulation of two opposing fibroblast trajectories

**Fig. 5:**
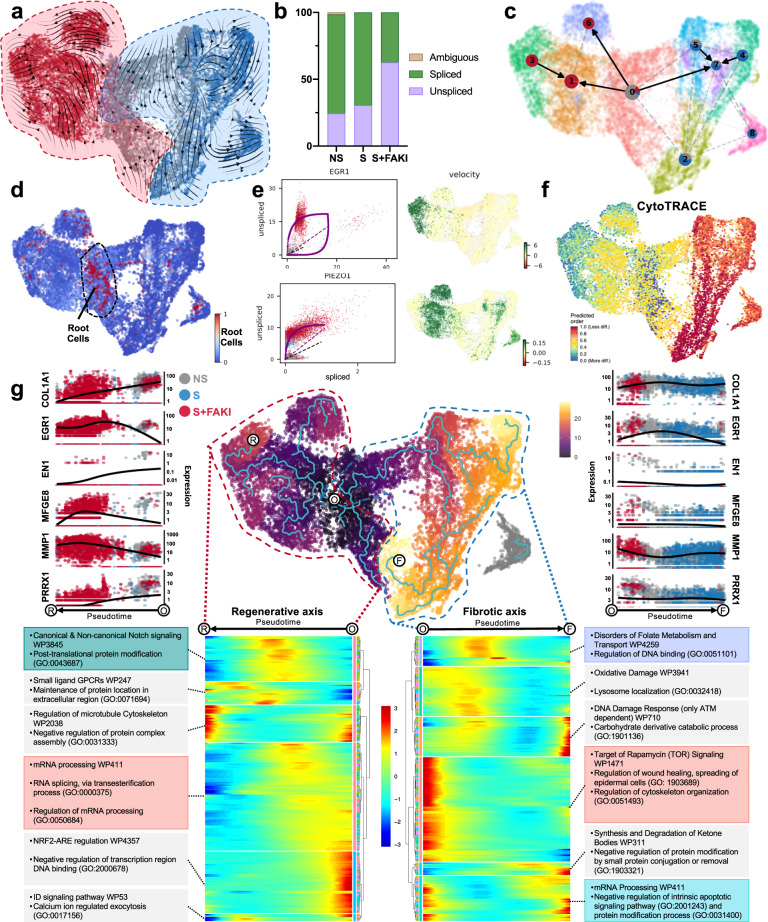
Mechanotransduction regulates opposing trajectories of profibrotic and regenerative fibroblast differentiation fates. **a** RNA velocities shown as the main gene-averaged flow, visualized by velocity streamlines projected onto the UMAP embedding. **b** Ratio of spliced to unspliced mRNA residuals in the three groups. **c** Partition-based graph abstraction (PAGA) showing the connectivity of cellular clusters with edge weights representing confidence in the presence of connections. **d** Root cells of cellular differentiation as identified by RNA velocity analysis. **e** Left: Gene-resolved velocities for *EGR1* and *PIEZO1*. The dotted line represents the estimated ‘steady-state’ ratio of unspliced to spliced mRNA abundance. RNA velocities are the residuals from the steady-state line, with positive velocities indicating an upregulation of a gene (i.e., a higher abundance of unspliced mRNA than expected in the steady state). Right: Gene-specific RNA velocity projected onto the UMAP embedding. **f** Cells colored by CytoTRACE scores. **g** Cells colored by pseudotime determined using the Monocle3 package. White dots delineate the root of trajectory origin (O), end point for the regenerative axis (R), and end point for the fibrotic axis (F). Expression of key marker genes is plotted over pseudotime along both regenerative (left) and fibrotic (right) trajectories. Bottom panel shows pseudotime heatmaps for regenerative (left) and fibrotic (right) trajectories and corresponding enriched genesets per heatmap cluster (Human WikiPathways and GO terms).

Traditional differential expression analysis (Fig. 4) only provides a snapshot of mRNA expression. We therefore employed RNA velocity analysis using the scVelo package to explore the comparative abundance of spliced and unspliced pre-mRNA transcripts in fibroblast clusters^64^. scVelo uses a dynamical likelihood-based model, which identifies velocity states and transcriptional dynamics of each individual cell in an unbiased manner (Supplementary Fig. 6a)^64,65^. Two opposing trajectories of fibroblast differentiation were identified in both the pooled and individual patient datasets, triggered either by the activation of mechanotransduction pathways or the disruption of mechanical signaling by FAK inhibition (Fig. 5a, Supplementary Fig. 7a). We found that FAK inhibition strongly increased the transcriptional activity of mechanically activated fibroblasts, resulting in a higher proportion of unspliced pre-mRNA, which accounted for 60% of all mRNA transcripts versus 30% in control and strained cells (Fig. 5b). To quantify the relationship between fibroblast clusters resulting from either mechanical activation or disruption of mechanotransduction, we applied partition-based graph abstraction (PAGA) informed by velocity-inferred directionality to quantify the relationship between fibroblast clusters resulting from either mechanical activation or disruption of mechanotransduction (Fig. 5c)^66^. The fibroblasts of the control group (cluster 0) were identified as the origin of the underlying Markov transition matrix, confirming their identity as root cells of fibroblast differentiation (Fig. 5d). Partition-based graph abstraction (PAGA) identified trajectory vectors pointing from the control fibroblasts toward either activated profibrotic clusters in response to mechanical strain (2, 4, 5, 7, 8) or regenerative clusters in response to mechanotransduction blockade (1, 3, 6) (Fig. 5c).

Using RNA velocity analysis, we identified several genes with differential proportions of unspliced to spliced mRNA among the treatment groups. Specifically, *EGR1* showed a high proportion of unspliced pre-mRNA in FAK-inhibited cells, providing further evidence for its role as a driver gene of regenerative fibroblast clusters (Fig. 5e). Additionally, *MDM2*, a gene regulated by AKT expression that regulates apoptosis, showed similar kinetics and further demonstrates how AKT-reliant genes persist within the FAK-inhibited fibroblast subset (Supplementary Fig. 4c)^67^. RNA velocity analysis also revealed an enrichment of pre-mRNA for *PIEZO1* and *CAPZA2* in FAK-inhibited cells. *PIEZO1* has been recently implicated in regulating mechanotransduction in macrophages^68^, and *PIEZO1* activation in fibroblasts may also mitigate fibroblast migration into the wound and contribute to the antifibrotic effects observed in response to FAK inhibition^69^. *CAPZA2* is an f-actin capping complex that binds to the barbed ends of actin filaments, preventing further addition of actin monomers. This reduces subsequent actin polymerization and may limit the ability to form a functional contractile myofibroblast phenotype in FAK-inhibited fibroblasts^70^. We also compared the relative differentiation states of individual cells based on the distribution of unique mRNA transcripts using CytoTRACE^71^. Mechanically stimulated fibroblasts appeared less differentiated compared to other cells due to a high number of uniquely expressed mRNA features, further demonstrating how strain initiates transcription of a wide range of unique profibrotic gene expression profiles in fibroblasts (Fig. 5f; Supplementary Fig. 7b).

To further understand the transcriptional shifts observed in our single-cell data, we constructed pseudotime trajectories^72^. Using the locus of origin identified by RNA velocity analysis (Fig. 5c, d), we found significant pseudotemporal divergence between mechanically strained, untreated fibroblasts and FAK-inhibited fibroblasts (Fig. 5g center; Supplementary Fig. 8a, b). These findings demonstrate that mechanotransduction alters fibroblast programming, and that blocking mechano-signaling in strained fibroblasts alters the resulting cellular program in a way that is transcriptionally similar to the native fibroblast base state of control cells. Using pseudotime analysis, we identified a regenerative axis leading from the control cells (origin, O) to the FAK-inhibited cell clusters (regeneration, R), and a fibrotic axis leading in the opposite direction toward the mechanically strained, untreated fibroblast clusters (fibrosis, F) (Fig. 5g, center; Supplementary Fig. 4b). These diverging trajectories were also observed with RNA velocity analysis and PAGA (Fig. 5a, c), with vectors radiating out of cluster 0 into either regenerative or fibrotic clusters. Along the fibrotic axis, we observed a transcriptional increase in the previously identified genes *COL1A1, PDGFRA, POSTN, RUNX1*, and *EN1*, which are hallmarks of myofibroblast proliferation, mechanotransduction, and collagen production (Fig. 5g; Supplementary Fig. 8a, b). This trajectory was also characterized by an induction of genesets for cell metabolism and regulation of DNA binding (GO:0051101), demonstrating an increasingly active and proliferative process (Fig. 5g, bottom right). In contrast, the trajectory from control to the FAK-inhibited cells (regenerative axis) exhibits a preservation of the AKT pathway (shown by *AKT1* expression), along with increased *EGR1*, *MFGE8, MMP1*, and *PIEZO1* expression. In contrast, myofibroblast markers, such as *PRRX1*, *POSTN, RUNX1, EN1*, and *PDGFRA,* clearly decreased along this axis (Fig. 5g; Supplementary Fig. 8a, b). Pathways, such as mRNA processing (WP411), RNA splicing (GO:0000375), and regulation of mRNA processing (GO:0050684), decreased along the regenerative trajectory (Fig. 5g, bottom left), correlating with our RNA velocity analysis. In summary, we utilized advanced bioinformatic tools to identify two diverging transcriptional trajectories, either toward fibrosis or toward regeneration. Along these opposing trajectories, we observed stark differences in transcriptional profiles that either defined fibrotic or regenerative fibroblast phenotypes.

### Porcine and human confirmation of diverging trajectories

**Fig. 6:**
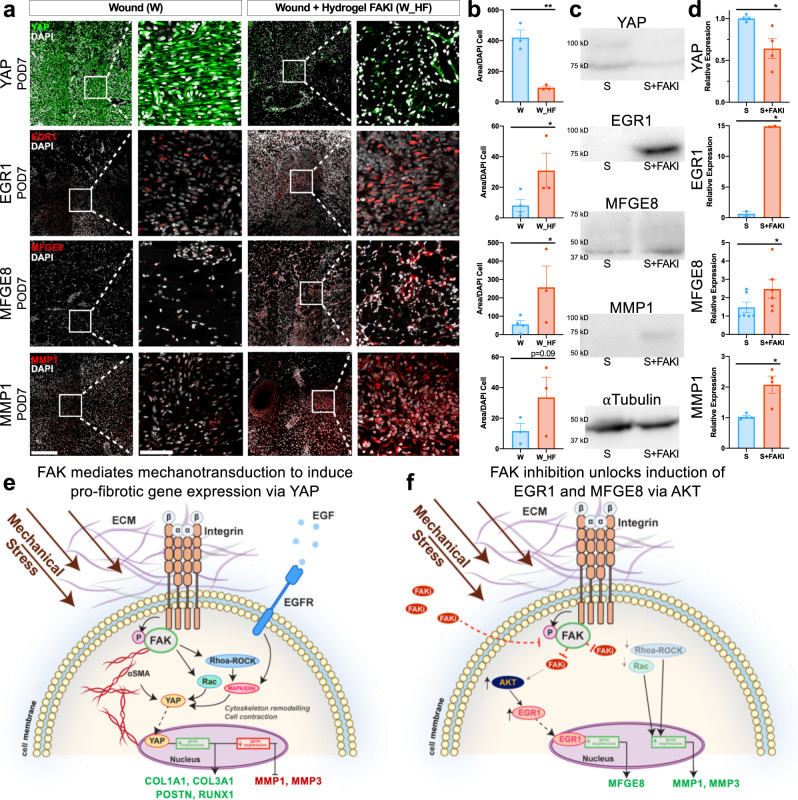
Protein level confirmation of human scRNA-seq observations in large animal comparator. **a, b** Protein level confirmation performed using immunofluorescence staining of wounded and untreated (W, left, blue, *n* = 3 independent wounds) vs. wounded and FAK-inhibited (W_HF, right, red, *n* = 3 independent wounds) porcine dermal tissue sections at POD7 (from Figs. 1, 2). Staining for YAP (encoded by the gene *YAP1;* **p = 0.0015), which contributes to myofibroblast differentiation, mechanotransduction, and scar formation, or EGR1 (*p = 0.0415), MFGE8 (*p = 0.0499), and MMP1, which contribute to regenerative healing and collagen degradation. Scale bar: 200 µm*.* Magnified image scale bar: 50 µm. **c, d** Western blot protein quantification of human fibroblasts from repeated experiments utilizing our collagen scaffold system of YAP1 (*n* = 4 independent collagen scaffolds per condition, *p = 0.0493), EGR1 (*n* = 2 independent collagen scaffolds per condition, *p = 0.0134), MFGE8 (*n* = 6 independent collagen scaffolds per condition, *p = 0.0164), and MMP1 (*n* = 4 independent collagen scaffolds per condition, *p = 0.0471). **e, f** Schematic of the proposed cellular mechanism of action showing how increased mechanical stress drives fibrosis and scar formation, while FAK inhibition unlocks AKT activation of EGR1 and MFGE8. Statistical comparisons were made using either unpaired (**b**) or paired (**d**) two-tailed *t*-tests. Each datapoint represents an independent wound or collagen scaffold. All data represent mean ± SEM. Representative images are shown from similar images across all experiments.

In order to confirm these diverging proregenerative and profibrotic differentiation trajectories as well as correlate human and large animal phenotypes (Figs. 1, 2), we first performed immunofluorescent staining of tissue sections from porcine wounds for the key regenerative and fibrotic markers identified in our human fibroblasts. FAK inhibition significantly blocked YAP expression at POD7 after injury (**p < 0.01) (Fig. 6a, b), while also promoting expression of top regenerative markers EGR1, MFGE8, and MMP1 (*p < 0.05) (Fig. 6a, b). These observations persisted at POD90 for both the fibrotic marker YAP and the regenerative marker EGR1, highlighting the long-lasting effects of alterations in mechanotransduction upon fibroblast phenotypes (Supplementary Fig. 9a, b). Expression of MMP1 and MFGE8 normalized by POD90, demonstrating that key ECM remodeling occurs early and tapers off by late timepoints (Supplementary Fig. 9c, d).

We then repeated our human fibroblast experiments to confirm these key markers on both the mRNA and protein level using qPCR and western blotting (Fig. 6c, d; Supplementary Fig. 10a). First, we confirmed that mechanical strain indeed induced fibrotic YAP mRNA and protein expression (Fig. 6c, d; Supplementary Fig. 10a). Mechanically strained fibroblasts treated with YAP1 small interfering RNA (siRNA) promoted *EGR1* expression and also decreased expression of *COL3A1* and *POSTN* (Supplementary Fig. 10b). These experiments demonstrated that YAP indeed acts as a master regulator of profibrotic differentiation, and that silencing YAP expression could, like FAK inhibition, promote regenerative *EGR1* expression while downregulating collagen and myofibroblast markers.

We also confirmed on the mRNA and protein levels that FAK inhibition increased EGR1, MFGE8, and MMP1 expression, while decreasing YAP and preserving AKT expression (Fig. 6c, d; Supplementary Fig. 10a). Silencing *EGR1* using siRNA, however, negated the beneficial effects of FAK inhibition by instead promoting profibrotic *YAP1* and *COL3A1* expression (Supplementary Fig. 10c). These findings confirmed that disruption of mechanotransduction unlocks a regenerative differentiation path driven by the master transcription factor *EGR1*.

Overall, these data strongly indicate the presence of two distinct profibrotic and proregenerative differentiation paths dependent on mechanotransduction signaling that occur after injury in both large animals and humans. On both the mRNA and protein levels, we confirmed the interlinked behavior of both YAP and EGR1 signaling and showed how silencing the expression of each of these master transcriptional factors directly pushes fibroblasts toward the opposing trajectory. Taken together, our findings identify a critical role of mechanical signaling in wound healing and scar formation for large organisms and highlight that true tissue regeneration can occur by blocking mechanical signaling.

## Discussion

Our study indicates that modulation of mechanotransduction can push and pull fibroblast programming either toward or away from fibrotic transcriptional states, highlighting the critical importance of mechanical forces in tissue regeneration. This principle has not been previously demonstrated in the context of large organisms, including humans. To our knowledge, this is the first study to demonstrate that inhibition of mechanotransduction through the FAK pathway improves scar formation, encourages skin regeneration, and promotes regenerative fibroblast phenotypes in a large animal porcine model and a physiologically relevant 3D human fibroblast culture system.

We demonstrate that high mechanical stress promotes heterogeneous myofibroblast differentiation and collagen deposition, leading to slower wound healing and eventual fibrosis (Fig. 6e). Inhibition of mechanotransduction through the FAK pathway blocks the appearance of profibrotic fibroblast subpopulations, accelerates wound healing, promotes skin regeneration, and reduces fibrosis, bringing the healed tissue almost back to a normal state. In human cells cultured in a physiologic three-dimensional system, we captured previously unknown cellular kinetics driving fibroblast differentiation during fibrotic processes. At a cellular level, we showed that inhibiting fibroblast mechano-sensation through FAK reduces profibrotic transcriptional signatures and interestingly preserves AKT signaling to create putatively regenerative fibroblasts characterized by *EGR1*, *MMP1*, and *MFGE8* expression (Fig. 6f)^53–55^. Furthermore, scRNA-seq analysis allowed us to frame these findings along an axis of fibrosis-regeneration, capable of modeling multigene contributions. Along this axis, we observed that mechanotransduction increases mRNA splicing and unique mRNA transcripts, contributing to increased fibroblast heterogeneity. Future work should be done to investigate the effects of mechanotransduction on other cell types, such as inflammatory infiltrates or other skin cells associated during wound healing (e.g., keratinocytes). While there is some recent evidence that myeloid cells respond to mechanical cues in certain situations, these studies have yielded conflicting results^73,74^. The effects of mechanotransduction on the crosstalk between fibroblasts and inflammatory or epithelial cells will further improve our ability to promote tissue regeneration after injury.

Collectively, our study represents the most comprehensive characterization linking mechanical forces and tissue regeneration in large organisms, including humans. We demonstrate that locally disrupting mechanical forces encourages true tissue regeneration in large organisms. These findings may have profound implications for future efforts to regenerate limbs, hearts, and other tissues.

## Methods

### Blank and FAKI-releasing pullulan-collagen hydrogel production

Blank and FAKI-releasing hydrogel patch production was conducted^11^. First, one gram of pullulan (TCI, Tokyo) was mixed with 1 g of trisodium trimetaphosphate (STMP) (Aldrich, St Louis, MO) and 1 g potassium chloride KCl (Fisher Scientific, USA). Deionized water was added to the powder mixture to a total volume of 5 mL and thoroughly mixed, followed by 5 mL of 10 mg/mL bovine collagen (Sigma–Aldrich, USA) suspension in 0.01 M hydrochloric acid (HCl). The resulting mixture was vortexed until a homogenous suspension was obtained, and then gently vortexed again after adding 0.65 mL of 1 N sodium hydroxide NaOH to initiate crosslinking. The mixture was then aliquoted in silicon molds and allowed to dry overnight in a sterile hood at room temperature. The dried films were washed with deionized water to remove non-crosslinked polymers, KCl, and NaOH until the pH of the wash solution was between 7.0 and 7.5. Swollen hydrogels were frozen at −80 °C followed by lyophilization to obtain dry (blank) patches.

FAKI (VS-6062) compound was obtained from Verastem Oncology (Needham, MA) and Selleckchem (Houston, TX). To incorporate FAKI in the hydrogels, we first dissolved FAKI in acetone at 1 mg/mL. One milliliter of the solution was spread uniformly upon the porous hydrogel, followed by the solvent evaporation in the hood. Blank and FAKI-containing hydrogel patches were placed in individual plastic bags and sterilized using e-beam irradiation at a 20kGy irradiation dose.

### Animal care

We have complied with all relevant ethical regulations for animal testing and research, and all animal work received ethical approval in accordance with the Administrative Panel on Laboratory Animal Care protocols (APLAC# 31530 and 32962) approved by Stanford University. Seven female red Duroc pigs, 6–8 weeks old and weighing ~16–20 kg at the time of surgery, were purchased from Pork Power Farms (Turlock, CA). All animals were acclimated for at least 1 week upon arrival. All animals were fed lab porcine grower diet and water ad lib.

### Porcine deep partial-thickness excisional wound model

Prior to operation, animals were administered oral amoxicillin 10 mg/kg for 24 h. General anesthesia was administered by Veterinary Services personnel and established with intramuscular telazol 6–8 mg/kg, administered once as a pre-anesthetic. Animals were then intubated using an endotracheal tube and maintained on 1.5–3% of inhaled isoflurane throughout the procedure. The hair on the back was clipped and skin was cleansed initially with Betadine^®^ solution following by a 70% alcohol rinse. Excisional wounds were created with a standard electric Zimmer dermatome (Zimmer Biomet, Warsaw, IN). Up to eight wounds, ~5 × 5 cm in size, were created on each lateral flank, with 3–5 cm intervals between wounds (Fig. 1a, b). Multiple dermatome passes were performed to create deep partial-thickness wounds of uniform 0.07 inch depth. The wounds were randomly assigned to receive either FAKI hydrogel (W_HF), blank hydrogel (‘placebo’, W_H), or no hydrogel (wounded control, W) (*n* = 6–9 wounds per condition). Animals were given oral amoxicillin 10 mg/kg post-operatively twice a day for 5 days total. Wound dressings, including FAKI hydrogel patches, were changed every other day for the first 3 weeks after initial injury until POD 21 (Fig. 1d, Supplementary Fig. 1d). Thereafter, dressings were changed twice per week until POD90. Animals were subject to short-term sedation for each dressing change.

### Wound closure, visual scar assessment, and viscoelastic analyses

Wounds were monitored photographically at each dressing change. Days to wound closure, defined as complete re-epithelialization without open wound area, were determined for each wound based on gross photographic assessment. Quantification of scar metrics were performed using a Visual Analog Scale (VAS) for five components (vascularity, pigmentation, observer comfort, acceptability, and contour) by a panel of four blinded scar experts. Total scores are calculated as a composite of all five scores; lower scores indicate improved scar appearance. A cutometer (Dual MPA 580, Courage + Khazaka Electronic, Köln, Germany) was used to evaluate the firmness and elasticity of the healing wounds at POD 60. Cutometer assessment is the gold standard to measure viscoelasticity in human patients. The cutometer measures the vertical deformation of the skin surface by applying negative pressure (suction) through a small circular diameter (8 mm probe). Deformation (suction) for two seconds followed by two seconds of relaxation (no suction) was applied three times and averaged. The elasticity ratio (ability for tissue to return back to original setpoint) was measured during the relaxation period (R2 metric)^75^.

### Histological analysis of collagen architecture

Specimens were harvested from the center of each wound at intermediate timepoints and at the end of the study, fixed in 4% paraformaldehyde, dehydrated, and then paraffin embedded. Masson’s Trichrome staining and Picrosirius Red staining were performed. Picrosirius Red-stained images were captured using polarized light microscopy (Leica DM5000 B upright microscope). Analysis of fiber alignment was performed on Picrosirius Red-stained images at ×40 magnification using the custom software MatFiber, an intensity-gradient-detection algorithm we have previously used to analyze overall alignment of collagen fibers and stress fibers from multiple samples^25,26^ as well as the open-source package CurveAlign^27^ (Fig. 2, Supplementary Fig. 2). The mean vector length (MVL) represents the strength of alignment and ranges from a value of 0 (completely random fiber alignment) to 1 (completely aligned fibers). The overall strength of alignment of the fibers were calculated following methodology previously published^26^. Quantification of individual collagen fiber parameters was performed using CT-FIRE (http://loci.wisc.edu/software/ctfire)^27,76^. CT-FIRE analyzes individual fiber metrics such as length, width, angle, curvature, localized fiber density, and the spatial relationship between fiber and the associated boundary. The average fiber parameters for each sample were used for statistical analysis.

### Statistical analyses

Statistical analysis was performed in Prism8 (GraphPad, San Diego, California) using paired or unpaired Student’s *t*-tests, as well as one-way or two-way analysis of variance (ANOVA) with Tukey’s multiple comparisons test. Data are presented as means ± SEM. *P* values of *p* < 0.05 were considered statistically significant. Principal component analysis (PCA) of fiber parameters was performed with centering to mean zero and scaling to unit variance using the *prcomp* function in R. Plots were generated with 95% confidence intervals using the *ggbiplot* and *pca3d* packages.

### Implantation toxicity tests

The pig was anesthetized with 2% isoflurane delivered at a rate of 2 L/min through a fitted nose cone. Control of pain was achieved by intramuscular administration of caprofen administered prior to surgery with a second dose given at 24 h and then every 24 h as needed. The dorsal neck was prepped and draped in the usual sterile fashion using chlorhexidine. Lidocaine 2% with epinephrine will be utilized to provide local anesthesia to the dorsal neck of the pig. Using a 15 blade scalpel, a 2–3 cm incision was made subcutaneously. Metzenbaum scissors were used to dissect a subcutaneous pocket away from the incision 2 × 2 cm in size. A piece of prewet FAK inhibitor hydrogel 2 × 2 cm or smaller in size was carefully inserted into the subcutaneous pocket. Hemostasis was ensured. The deep dermal layer was closed with 4-0 vicryl suture using buried simple interrupted sutures. The skin was closed using 5-0 monocryl suture in a running subcuticular fashion. Dermabond skin glue was used to seal the incision. The skin was cleaned with wet and dry gauze and a sterile occlusive dressing was placed over the wound. The pig was monitored carefully in the postoperative period. The implanted hydrogel was kept for up to 4 weeks and local parameters (edema, erythema, bleeding, skin peeling or ulcers) were monitored daily. After 4 weeks, we obtained an excisional 8 mm tissue specimen using a biopsy tool.

### Immunofluorescent staining

Immunofluorescent staining was performed using primary antibodies targeting EGR1 (1:100 dilution; ThermoFisher, PA5-83115), MFGE8 (1:100 dilution; ThermoFisher, PA5-82036), MMP1 (1:100 dilution; Abcam, ab52631), α-smooth muscle actin (1:200 dilution; Abcam, ab5694), YAP (1:100 dilution; CellSignaling, 14074 S), and Perilipin-1 (1:100 dilution; Abcam, ab172907). The percentage of fluorescent area was quantified using custom a MATLAB image processing code written by the authors and previously published^26^. All histology and immunofluorescent images shown are representative images of multiple experiments.

### Collection of human skin tissue

We have complied with all relevant ethical regulations for work with human tissue, and all human skin was collected with ethical approval under the IRB #54225 from Stanford University. Skin tissue was collected from patients undergoing procedures, which normally involve tissue removal (including plastic surgery operations). No patient identifying information was collected. This skin would otherwise be discarded as medical waste. Thus, there was no additional risk to patients and recruitment was not needed.

### Fibroblast-populated 3D collagen scaffold experiments

We isolated and cultured dermal fibroblasts from human skin samples from three surgical procedures: a breast mastectomy, an abdominoplasty, and a thighplasty (*n* = 3 patients). Fibroblasts were isolated by mechanical and enzymatic digestion and cultured under standard conditions until passage 3. The primary fibroblast cultures were then used to create fibroblast-populated collagen hydrogels at final concentration of 200k cells/mL and 2 mg/mL collagen (PureCol, Advanced Biomatrix, San Diego, CA), following our previously published protocols^26^. In brief, collagen scaffolds were formulated in a cruciform shape in petri dishes with a PDMS coating (~4 mm) on the bottom. Pins were pushed through the hydrogel cruciform arms to constrain the scaffolds in both directions for a 24 h preculture period before being subjected to either no strain, 10% equibiaxial strain, or strain + FAKI treatment for an additional 48 h. FAKI treatment was administered by adding 20 mM FAKI in DMSO into the culture media of the scaffolds to achieve a final concentration of 10 μM FAKI for 48 h. Strained but untreated fibroblasts were also treated with the same volume of DMSO only. Strain was imposed by removing the anchoring pins, manually extending the hydrogel cruciform arms, and pushing the pins back to hold the arms in the new, extended position. We applied nine Titanium(IV) oxide paint dots (Sigma–Aldrich) on the surface of the central region of the gel to track and quantify the imposed strains. We used a digital camera to image the markers before and after strain. Photographs of marker position were used to compute a single homogenous deformation gradient tensor **F** that provided the least-squares best fit mapping of the 9 marker positions from the undeformed to deformed positions by solving the overdetermined matrix equation:

1 $${{{{{\boldsymbol{x}}}}}}={{{{{\boldsymbol{FX}}}}}}+{{{{{\boldsymbol{p}}}}}},$$

where **p** is an arbitrary vector included to account for translation between images. We converted the deformation to a strain tensor **E** using:

2 $${{{{{\boldsymbol{E}}}}}}=\frac{{{{{{\bf{1}}}}}}}{{{{{{\bf{2}}}}}}}\left([{{{{{\boldsymbol{F}}}}}}^{{{{{\boldsymbol{T}}}}}}{{{{{\boldsymbol{F}}}}}}]^{{{{{\bf{2}}}}}}-{{{{{\boldsymbol{I}}}}}}\right)$$

### Single-cell barcoding, library preparation, and single-cell RNA sequencing

After 2 days of increased (induction of strain) or inhibited (induction of strain + FAKI) mechanotransduction, collagen scaffolds were micro-dissected and enzymatically digested in a 50 mL conical tube containing 20 mL Collagenase, Type I (ThermoFisher) in PBS at a concentration of 5 mg/mL for enzymatic digest^35^. The cell-digest suspensions were constantly agitated (rotated) for a total of 1 h at 37 °C. The sample was then subjected to maximum speed on a vortex mixer (VWR) for 30 seconds to physically disrupt any tissue that had clumped together and thus maximize the tissue surface area exposed to enzymatic digestion at all times. The cellular and enzymatic solution was then pipetted through a 100 µm Nylon cell filter (Fisher-Scientific) into a new conical tube, and 20 mL of 10% FBS DMEM was added through the filter to quench the enzymatic reaction and release any cells trapped within the filter, maximizing downstream cell yield. Solutions were then spun at 300 × *g* for 8 min at 4 °C in a centrifuge to pellet the cells, resuspended in 20 mL 10% FBS DMEM, and passed through a 70 µm Nylon cell filter. A 20 mL solution of 10% FBS PBS (FACS Buffer) was added through the filter to collect the remaining cells.

To remove excessive debris left by the collagen hydrogel and dead cells, we stained the cellular suspension with Propidium Iodide (biolegend) and sorted this cellular suspension for live cells at the Stanford Shared FACS Facility (Supplementary Fig. 11). The sorted cells were resuspended to a final cellular concentration of 1200 cells/µL in 0.04% Bovine Serum Albumin (BSA; Sigma–Aldrich) in PBS in accordance with the maximum capture concentration short of overloading, per specifications from 10× Genomics (Pleasanton, CA). This cellular suspension was then submitted for droplet-based microfluidic single-cell RNA sequencing (scRNA-seq) at the Stanford Functional Genomics Facility (SFGF) using the 10x Chromium Single Cell platform (Single Cell 3’ v3, 10× Genomics, USA). A droplet of the cell suspension, reverse transcription master mix, and partitioning oil was loaded onto a single-cell chip and processed on the Chromium Controller. Reverse Transcription was performed at 53 °C for 45 min. cDNA was amplified for 12 cycles total (BioRad C1000 Touch thermocycler) with cDNA size selected using SpriSelect beads (Beckman Coulter, USA) and a 0.6 ratio of SpriSelect reagent volume to sample volume. cDNA was analyzed on an Agilent Bioanalyzer High Sensitivity DNA chip for qualitative control purposes. cDNA was fragmented using the proprietary fragmentation enzyme blend for 5 min at 32 °C, followed by end repair and A-tailing at 65 °C for 30 min. cDNA were double-sided size selected using SpriSelect beads. Sequencing adaptors were ligated to the cDNA at 20 °C for 15 min. cDNA was amplified using a sample-specific index oligo as primer, followed by another round of double-sided size selection using SpriSelect beads. Final libraries were analyzed on an Agilent Bioanalyzer High Sensitivity DNA chip for qualitative control purposes. cDNA libraries were sequenced on a HiSeq 4000 Illumina platform aiming for 50,000 reads per cell.

### Data processing, FASTQ generation, and read mapping

Base calls were converted to reads using the Cell Ranger (10× Genomics; version 3.1) implementation of *mkfastq* and then aligned against the GRCh38 v3.0.0 (human) genome using Cell Ranger’s count function with SC3Pv3 chemistry and 5000 expected cells per sample^77^. Cell barcodes representative of quality cells were delineated from barcodes of apoptotic cells or background RNA based on a threshold of having at least 200 unique transcripts profiled, less than 10,000 total transcripts, and less than 10% of their transcriptome of mitochondrial origin^78^. We also considered evaluating stricter thresholds (e.g., at least 500 unique transcripts), but found that cells falling between 200 and 500 unique transcripts exhibited profiles consistent with intact cells, notably with lower percent mitochondria RNA.

### Data normalization and cell subpopulation identification

Unique molecular identifiers (UMIs) from each cell barcode were retained for all downstream analysis. Raw UMI counts were normalized with a scale factor of 10,000 UMIs per cell and subsequently natural log transformed with a pseudocount of 1 using the R package Seurat (version 3.1.1)^79^. Highly variable genes were identified, and cells were scaled by regression to the fraction of mitochondrial transcripts. Aggregated data were then evaluated using uniform manifold approximation and projection (UMAP) analysis over the first 15 principal components^80^. Automated cell-level annotations were ascribed using the SingleR toolkit (version 3.11) against the ENCODE blue database^81^.

### Generation of characteristic subpopulation markers and enrichment analysis

Cell-type marker lists were generated with Seurat’s native *FindMarkers* function with a log fold-change threshold of 0.25 using the ROC test to assign predictive power to each gene. The 100 most highly ranked genes from this analysis for each cluster were used to perform geneset enrichment analysis against pathway databases in a programmatic fashion using EnrichR (version 2.1)^82^.

### Over-representation analysis using Genetrail3

Using GeneTrail v3.0^42^, an over-representation analysis (ORA) was performed for each cell using the 500 most expressed protein coding genes with the genesets *Gene Ontology: Biological Process* (GO-BP) and *WikiPathways* (WP). *P* values were adjusted using the Benjamini–Hochberg procedure and genesets were required to have between 2 and 1000 genes.

### CytoTRACE analysis

The CytoTRACE algorithm was used with default parameters to compare cellular differentiation states among fibroblasts in our dataset^71^. CytoTRACE analyzes the number of uniquely expressed mRNA features per cell, as well as other factors such as distribution of mRNA content, to calculate a score assessing the differentiation and developmental potential of cells.

### RNA velocity analysis using scVelo

RNA velocity analysis was performed using the dynamical model of the scVelo (v0.2.3) package^64^. Partition-based graph abstraction (PAGA) was performed using the *sc.tl.paga* function in scVelo. To find genes with differentially regulated transcriptional dynamics compared to all other clusters, a Welch *t*-test with overestimated variance to be conservative was applied using the *sc.tl.rank_velocity_genes* function. Genes were ranked by their likelihood obtained from the dynamical model grouped by treatment.

### Pseudotime analysis using Monocle

Monocle3 (v0.2.1.2) was applied to construct pseudotime trajectories for individual cells along their aggregate spatial manifold. Cell transcriptional states were first projected onto a reduced-dimensional space in a manner to minimize information loss using a modified UMAP implementation^83^. The Louvain community detection algorithm was then used to group mutually similar cells, and nearby groups were categorized into ‘supergroups’^84,85^. The paths and trajectories of individual cells and the locations of branches were then resolved within each supergroup^86^. To create pseudotime heatmaps, we created subsets of the regenerative or fibrotic trajectory cells and assigned pseudotime values to each subset. We used the function *graph_test* to identify the 200 genes with the strongest correlations with pseudotime and plotted their expression along pseudotime using Monocle2.

### Western blotting

Protein isolation, quantification, and western blot analysis were performed on fibroblasts within collagen scaffolds^87^. The scaffolds were minced with scissors and then combined with a solution of cold RIPA buffer (Sigma–Aldrich) containing 1 mM Phenylmethanesulfonyl Fluoride (PMSF; Cell Signaling) and Proteases Inhibitor Cocktail (Sigma–Aldrich). Following repeated freeze-thaw cycles, vortexing, and sonication, samples were centrifuged, and the supernatant was collected for protein quantification and western blot analysis. Each total protein sample was prepared for loading with NuPAGE LDS sample buffer and NuPAGE reducing agent according to manufacturer’s instructions (NuPage, Life Technologies). Protein samples were electrophoresed on NuPAGE Bis-Tris precast polyacrylamide gels (ThermoFisher) and transferred onto PVDF membrane (Immunoblot, Biorad, CA). Immunoblotting analysis was performed using primary rabbit antibodies of YAP (1:200 dilution; Cell Signaling, 4912), EGR1 (1:500 dilution; Cell Signaling, 4153), MFGE8 (1:500 dilution; Sigma–Aldrich, SAB1408603), MMP1 (1:1000 dilution; abcam, ab38929), and alpha-Tubulin (1:1000 dilution; Cell Signaling, 2125). A horseradish peroxidase-conjugated secondary anti-rabbit was used (1:2000 dilution; Cell Signaling, 7074). Immunoblotted proteins were visualized by enhanced chemiluminescence western blot reagents (Sigma–Aldrich) and film (Amersham Biosciences). Densitometry analysis of electrophoretic bands was performed using the ImageJ software program (NIH) and normalized to the loading control (alpha-tubulin). All western blot images shown are representative of multiple experiments.

### Realtime PCR (qPCR)

We performed qPCR using validated and predesigned TaqMan Gene Expression Assays (Sigma, 4331182) to quantify GAPDH (Hs02786624_g1), MMP1 (Hs00899658_m1), EGR1 (Hs00152928_m1), YAP1 (Hs00217433_m1), AKT1 (Hs00178289_m1), MFGE8 (Hs00983890_m1), COL3A1 (Hs00943809_m1), and POSTN (Hs01566750_m1) (Supplementary Table 1). We used a total reaction volume of 20 μl that contained 10 μl of TaqMan Universal Master Mix II, no UNG (Thermo Fisher Scientific, 4444963), 0.2 μM forward and reverse primers, 0.25 μM hydrolysis probe, and the appropriate amount of genomic DNA (40 ng per reaction), on an Applied Biosystems 7900 instrument (Thermo Fisher Scientific). SuperScript cDNA Synthesis Kit was used (Thermo Fisher Scientific, 11754050). qPCR conditions were 1 cycle of 95 °C for 10 min, followed by 50 cycles of 95 °C for 15 s, 56 °C for 30 s, and 72 °C for 30 s. All samples were run as triplicates.

### Small interfering RNA (siRNA)

We performed siRNA experiments according to established protocols. Briefly, subconfluent cells were transfected with a combination of (A) 8 μL siRNA duplex in 100 μL siRNA transfection medium (Santa Cruz Biotechnology, sc-36868) with (B) 8 μL siRNA transfection reagent (Santa Cruz Biotechnology, sc-29528) in 100 μL siRNA transfection medium. For siRNA duplex, we used either control (scrambled) siRNA (Santa Cruz Biotechnology, sc-37007), YAP1 siRNA (Santa Cruz Biotechnology, sc-38637), or EGR1 siRNA (Santa Cruz Biotechnology, sc-29303). Cells were incubated with this siRNA transfection reagent mixture for 7 h before normal growth medium containing 2× the normal serum and antibiotic concentrations were added. After an additional 24 h, cells were then used for collagen hydrogel scaffold experiments.

### Reporting summary

Further information on research design is available in the Nature Research Reporting Summary linked to this article.

## Supplementary information

Supplementary Information

Reporting Summary

## Data Availability

The authors declare that the source data supporting the findings of this study are provided with the manuscript and supplementary information files. The scRNA-seq data discussed in this publication have been deposited in NCBI’s Gene Expression Omnibus^88^ and are accessible through GEO Series accession number GSE167339. Automated cell-level annotations were ascribed using the SingleR toolkit (version 3.11) against the ENCODE blue database^81^. All other relevant data are available from the corresponding author on reasonable request. Source data are provided with this paper.

## Source data

Source Data

## Code availability

MatFiber code may be found on GitHub at https://github.com/cardiacbiomechanicsgroup/MatFiber. All other relevant code are available from the corresponding author on reasonable request.
