# Supplementary material for: Disrupting biological sensors of force promotes tissue regeneration in large organisms
Source: Nat Commun. 2021 Sep 6;12:5256. doi: 10.1038/s41467-021-25410-z (PMC8421385; doi:10.1038/s41467-021-25410-z)
Supplement: Supplementary file 1 — Supplementary Information [file 41467_2021_25410_MOESM1_ESM.pdf]

<u>Supplementary Data</u>: Disrupting Biological Sensors of Force Promotes Tissue Regeneration in Large Organisms

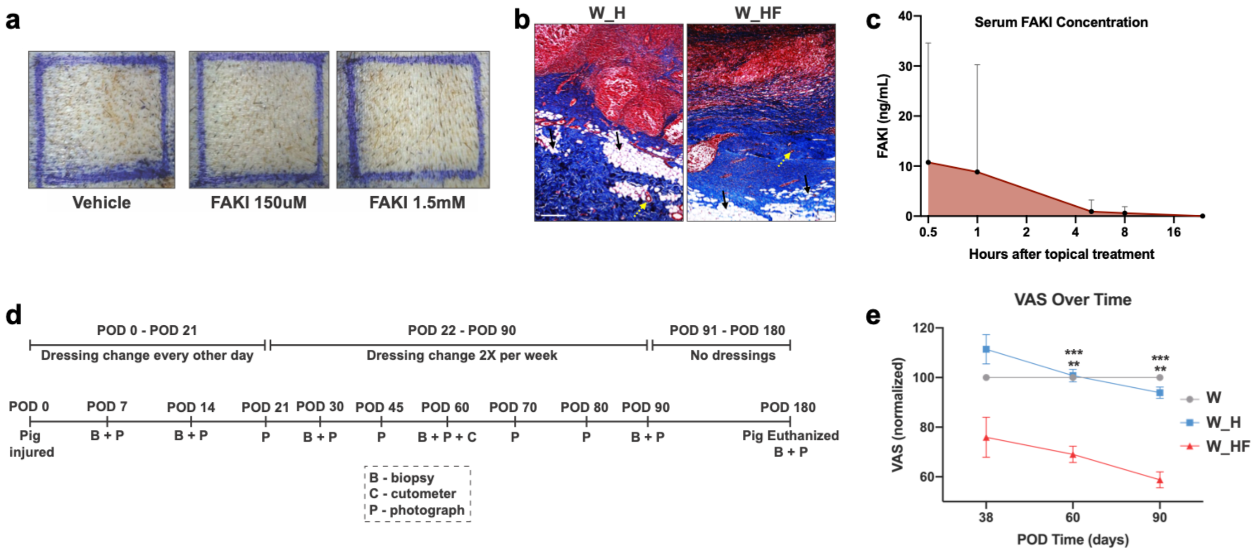

Supplementary Figure 1. Acute, systemic, and implantation toxicity testing of FAKI hydrogel in the porcine model. (a) To assess acute toxicity, concentrated FAKI solution (150 μM and 1.5 mM dissolved in 4% DMSO and 30% PEG-300) was dripped on unwounded pig skin daily for 14 consecutive days. Wounds were monitored by gross photography, and no adverse skin reactions were observed. Blue box size=25 cm^2. **(b)** 4 cm^2 size FAKI (W_HF) and blank hydrogels (W_H) were implanted into a subcutaneous pocket and closed using sutures for 14 days. Trichrome staining shows anatomic preservation of dermal and sub-dermal structures, demonstrating biocompatibility and lack of adverse reactions. *Data represent one experiment. Scale bar: 0.5mm*. **(c)** Peripheral blood samples from pigs treated with topically applied FAKI (covering approximately 7-10% of total body surface area) were analyzed with mass spectrometry to determine effect on serum FAKI levels. Measured serum levels were found to be only 1% of the maximum tolerated dose found in a human study of oral FAKI administration (1000 ng/mL)[1]. *Data collected from three independent experiments collected from three different pigs (n=3 independent experiments).* **(d)** Full schedule of events from the time of initial injury to POD180 is shown in two separate timeline diagrams. B – biopsy, C – cutometer reading, P – photograph. **(e)** Visual Analog Scale (VAS) scar scores of porcine deep partial-thickness wounds over time at POD38 (n=3 independent blinded scores), POD60 (n=4 independent blinded scores; W vs W_H: ***p=0.0052;* W_H vs W_HF ****p=0.0009*) and POD90 (n=4 independent blinded scores; W vs W_H: ***p=0.0022;* W_H vs W_HF ****p=0.0005*). *Each data point represents an independent, blinded VAS analysis*. *Statistical comparisons were made using a two-way analysis of variance (ANOVA) with Tukey's multiple comparisons tests. All data represent mean ± SEM.*

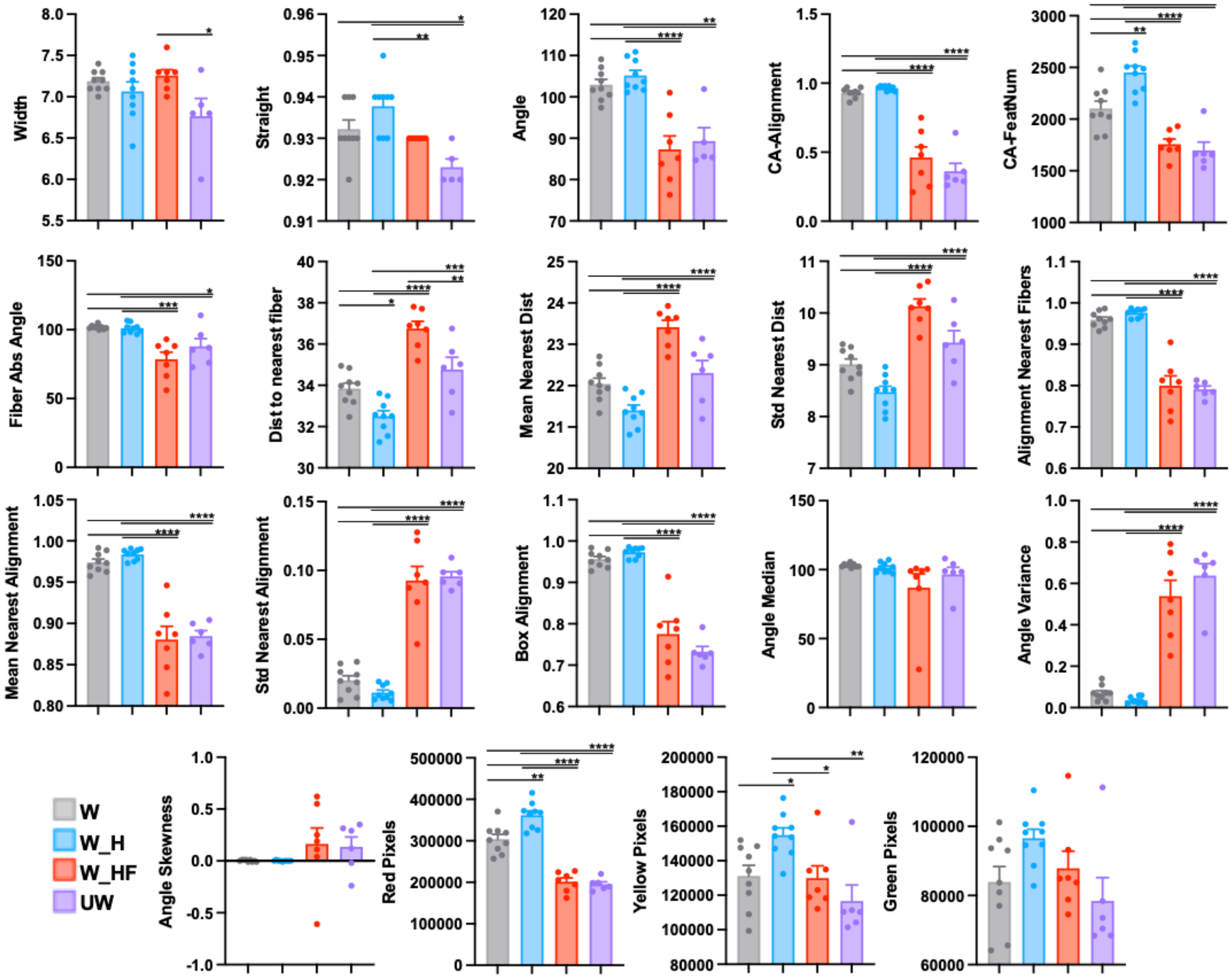

Supplementary Figure 2. Fiber analysis was performed using three established collagen analysis algorithms. Processed images visualizing fibers for quantification of picrosirius red-stained images performed using three previously published algorithms: MatFiber, CT-FIRE, and CurveAlign[2-5]. The 19 other collagen structural metrics are plotted here. Analysis across all metrics consistently demonstrated that FAKI restored properties closer to that of unwounded skin. These metrics were used together to calculate a principal component analysis seen in Figure 2c. Standard wounds (W, red, n=12 independent wounds), blank pullulan-collagen hydrogel treated wounds (W_H, blue, n=12 independent wounds), FAKI-releasing hydrogel treated wounds (W_HF, red, n=10 independent wounds), and unwounded skin (UW, purple, n=9 independent wounds). *Statistical comparisons were made using a one-way analysis of variance (ANOVA) with Tukey's multiple comparisons tests (*p<0.05, **p<0.01, ***p<0.001, ****p<0.0001). Each data point represents an independent wound. All data represent mean ± SEM.*

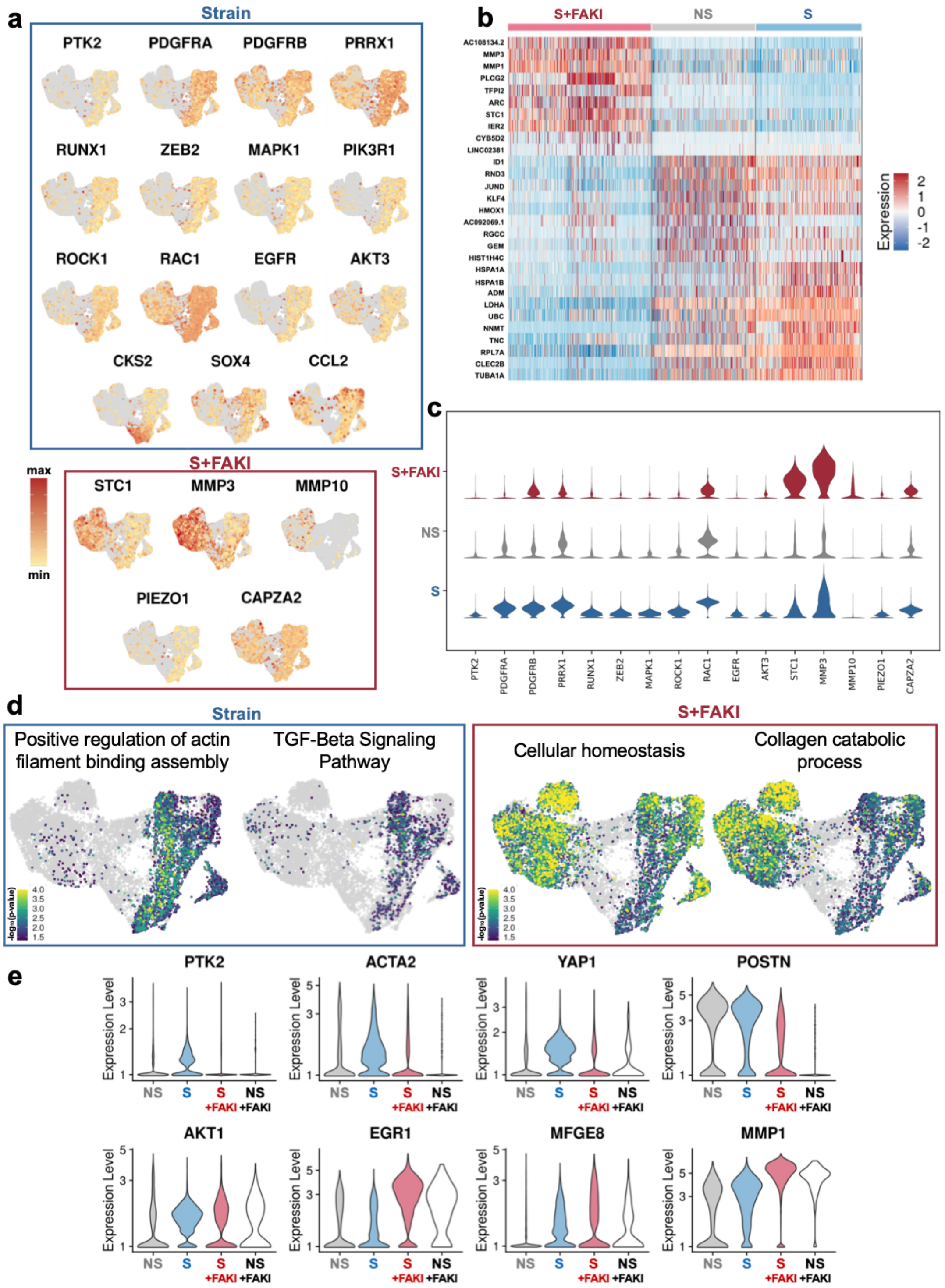

Supplementary Figure 3. Adult human dermal fibroblasts were seeded into 3D collagen scaffolds, and subjected to either no strain (NS, grey), strain (S, blue), strain with 10µM FAKI (S+FAKI, red), or no strain with FAKI (NS+FAKI, white) and then submitted for 10X genomics. (a) Select gene feature UMAP plots illustrating the expression levels. (**b**) Heatmap of the top 10 differentially expressed genes by treatment group. **(c)** Violin plots of cluster-defining differentially expressed genes. **(d)** GeneTrail feature UMAP plot of key pathways that differentiate the groups. **(e)** Violin plots comparing the main treatment groups with additional NS+FAKI group.

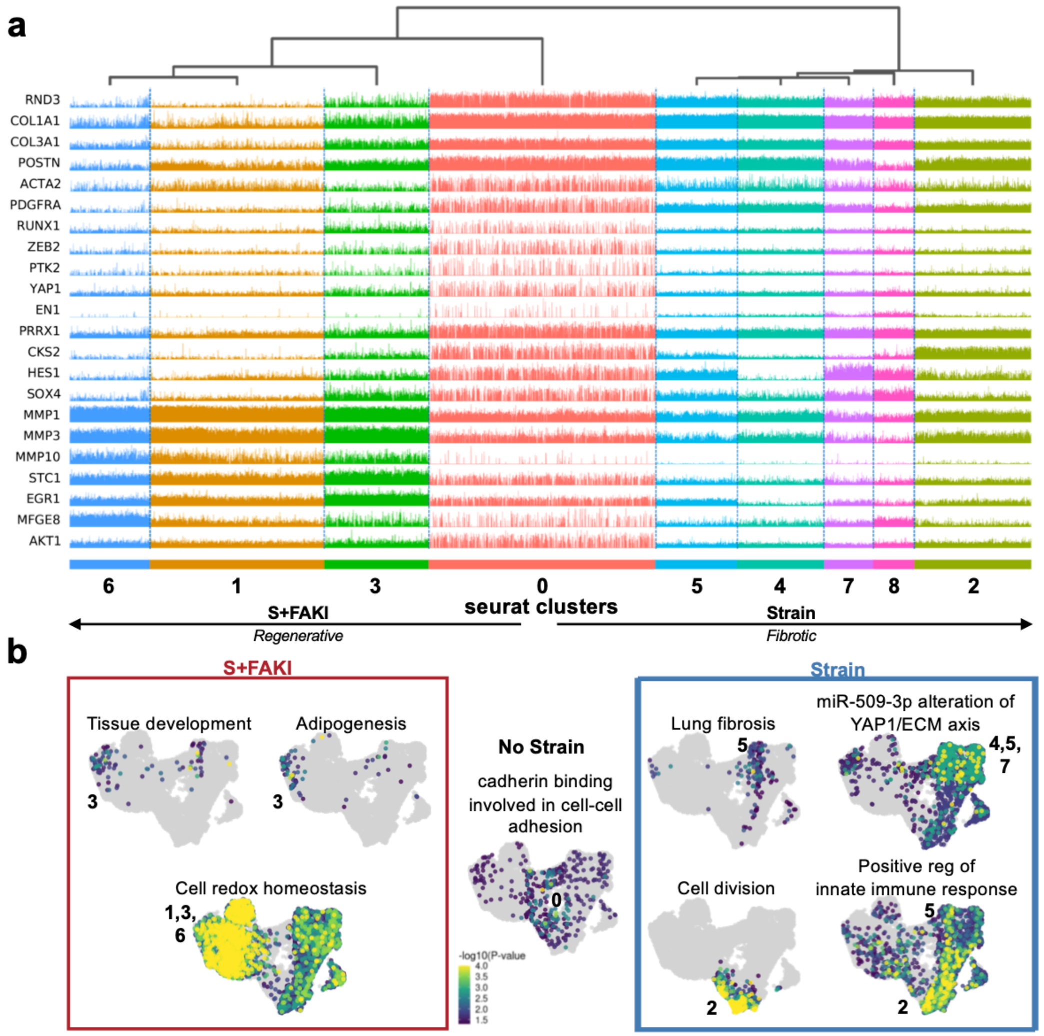

Supplementary Figure 4. Fibroblast cluster heterogeneity. (a) Expression of select genes for each cell within our dataset. Each cell's gene expression level is shown as a bar graph and divided by cluster. Dendrogram (top) groups similar clusters together. **(b)** GeneTrail feature UMAP plots of key pathways that differentiate each cluster (cluster number next to each plot).

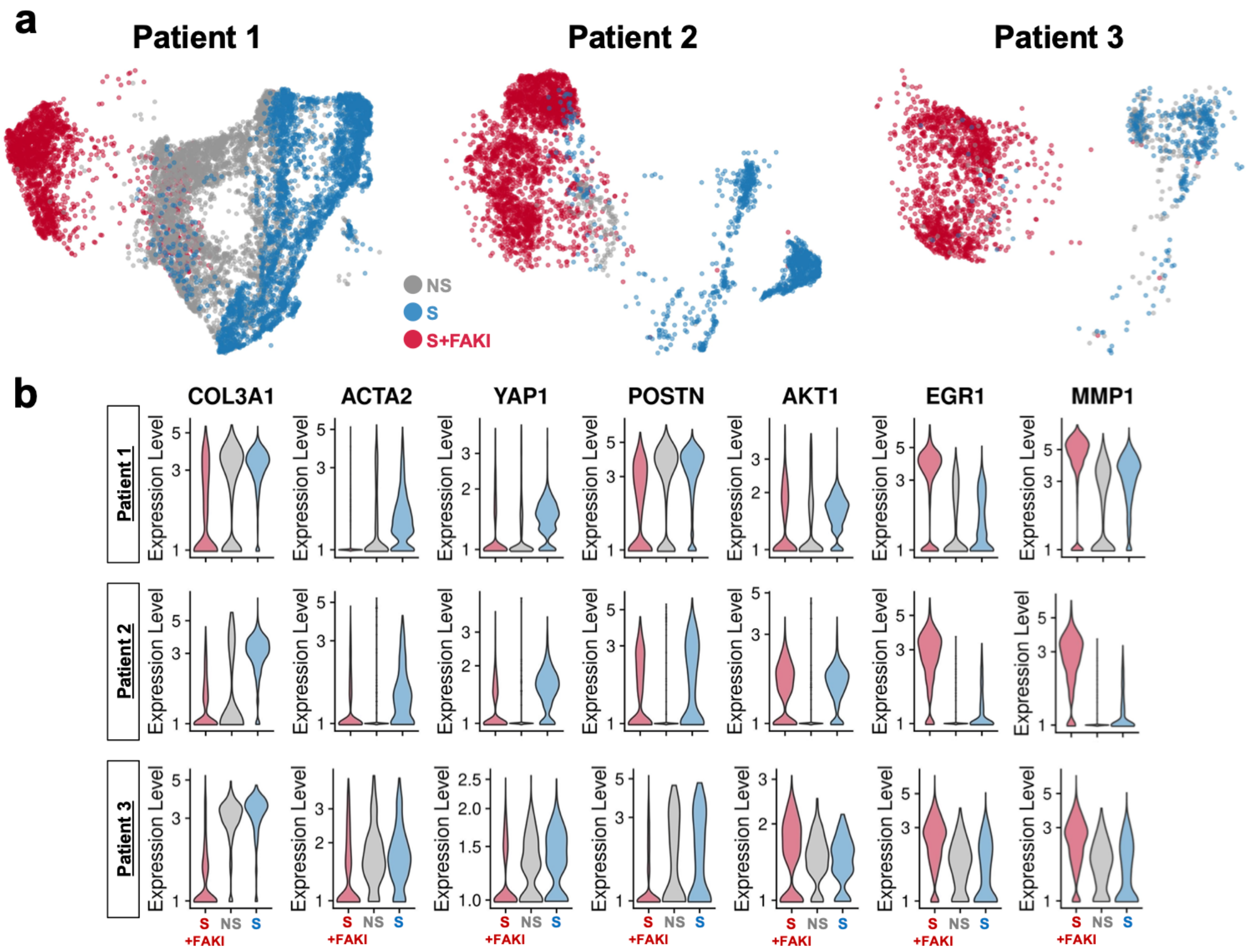

Supplementary Figure 5. Expression of genes of interest were consistent throughout the three human patients. (a) Combined UMAP embedding colored by each patient and their three experimental groups. **(b)** Violin plots of genes of interest, separated by each of the 3 patients. No strain (NS, grey), strain (S, blue), and Strain+FAKI (S+FAKI, red).

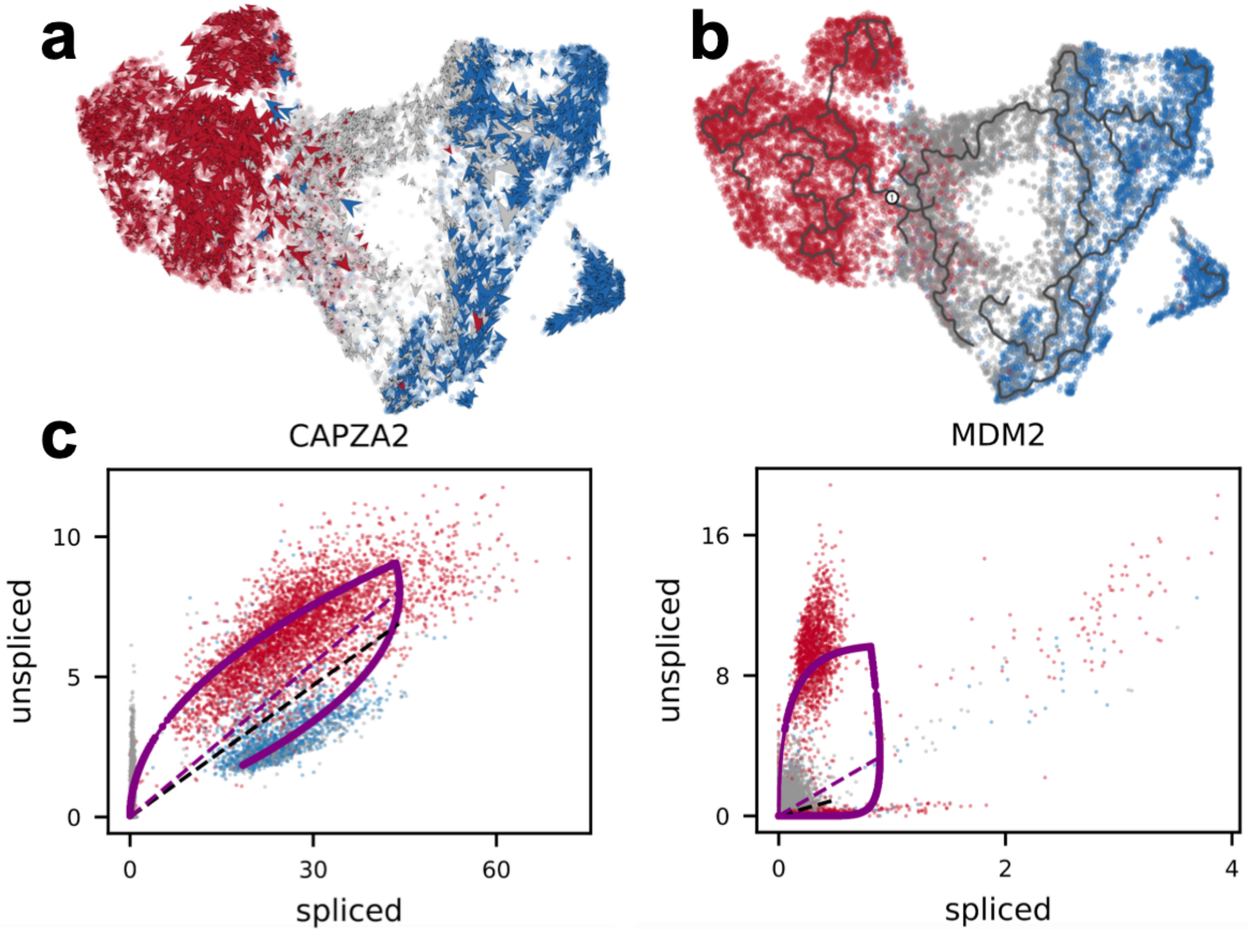

Supplementary Figure 6. Additional RNA velocity and Monocle3 analysis. (a) RNA velocities for each singular cell derived from the dynamical model of the scVelo package projected onto the UMAP embedding. Each cell is visualized with a velocity direction and velocity strength (size of cell). **(b)** Monocle3 derived cell trajectory plotted on UMAP embedding colored by treatment group. **(c)** Gene-resolved velocities for *CAPZA2* and *MDM2*. The dotted line represents the estimated 'steady-state' ratio of unspliced to spliced mRNA abundance. RNA velocities are the residuals from the steady-state line, with positive velocities indicating an up-regulation of a gene, i.e., a higher abundance of unspliced mRNA than expected in the steady state.

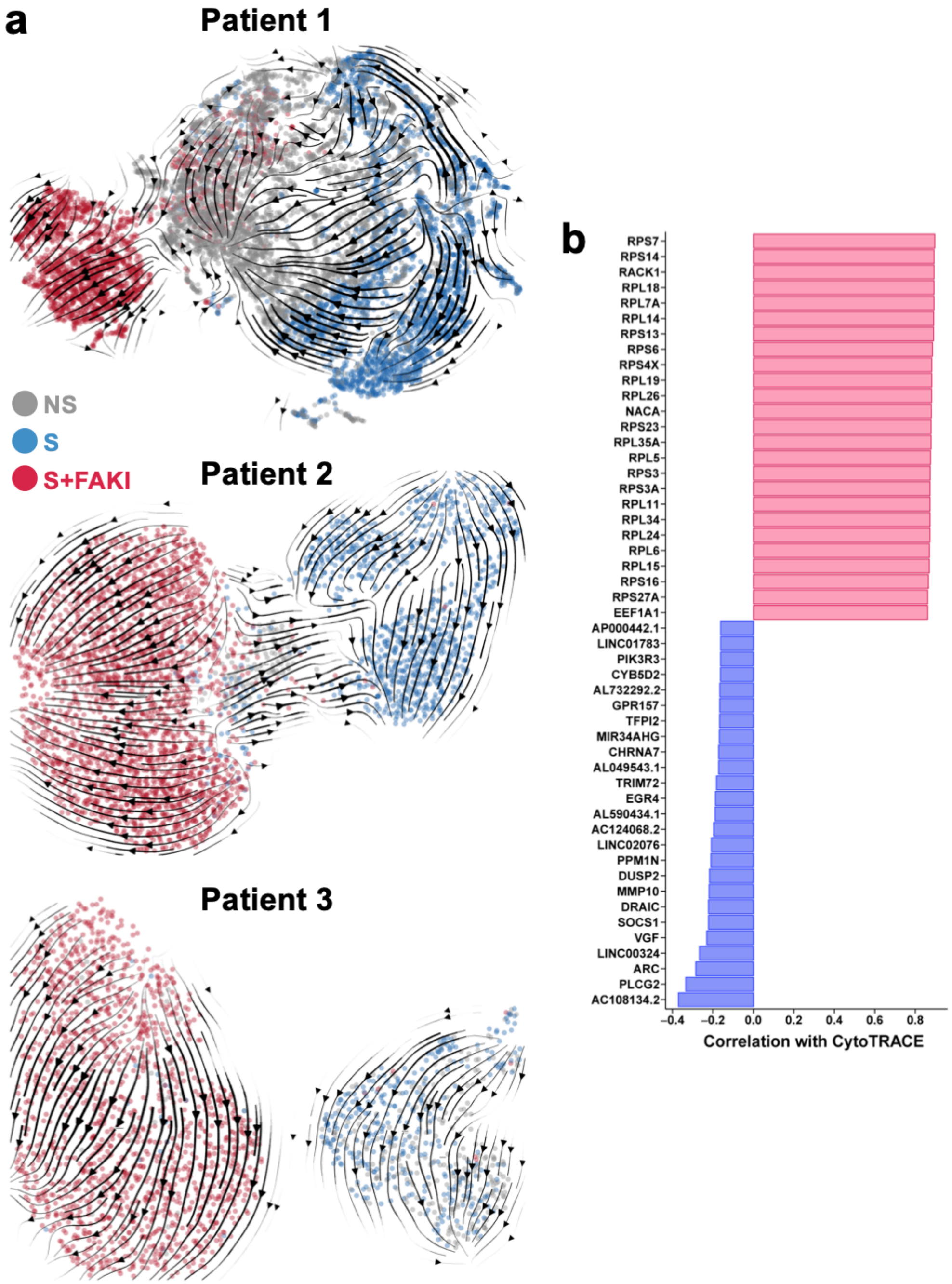

Supplementary Figure 7. (a) RNA velocity analysis according to fibroblasts from each of the three patients (patient 1, 2, and 3; shown in Figure 4a,b). This was derived from the dynamical model of the scVelo package and projected onto each patient's UMAP fibroblast embedding. The main gene-averaged flow is visualized by velocity streamlines. **(b)** Waterfall plots showing genes positively (red) and negatively (blue) correlating with CytoTRACE scores for cells in Figure 5f.

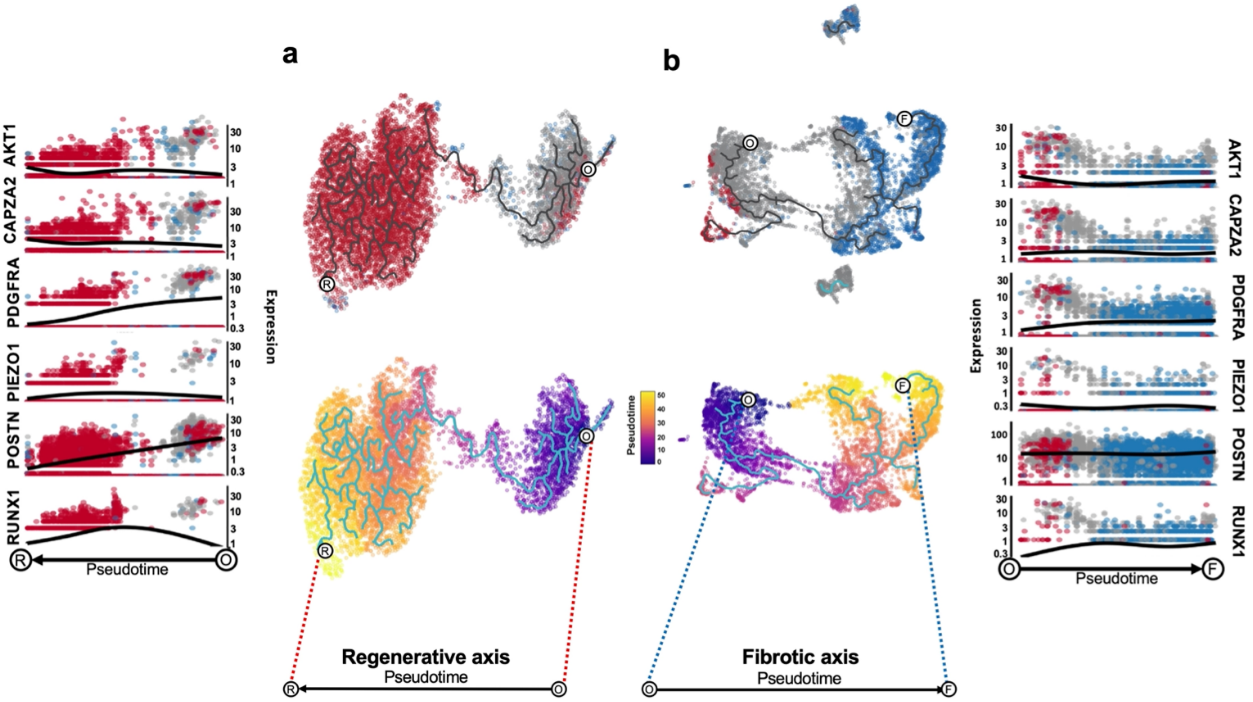

Supplementary Figure 8. (a,b) Cells colored by pseudotime determined using the Monocle3 package, separated by the **(a)** regenerative axis and **(b)** fibrotic axis. Circles show the root of origin (O), end point for the regenerative axis (R), and end point for the fibrotic axis (F). Gene pseudotime trajectory plots show gene expression along pseudotime for either the regenerative or fibrotic axes.

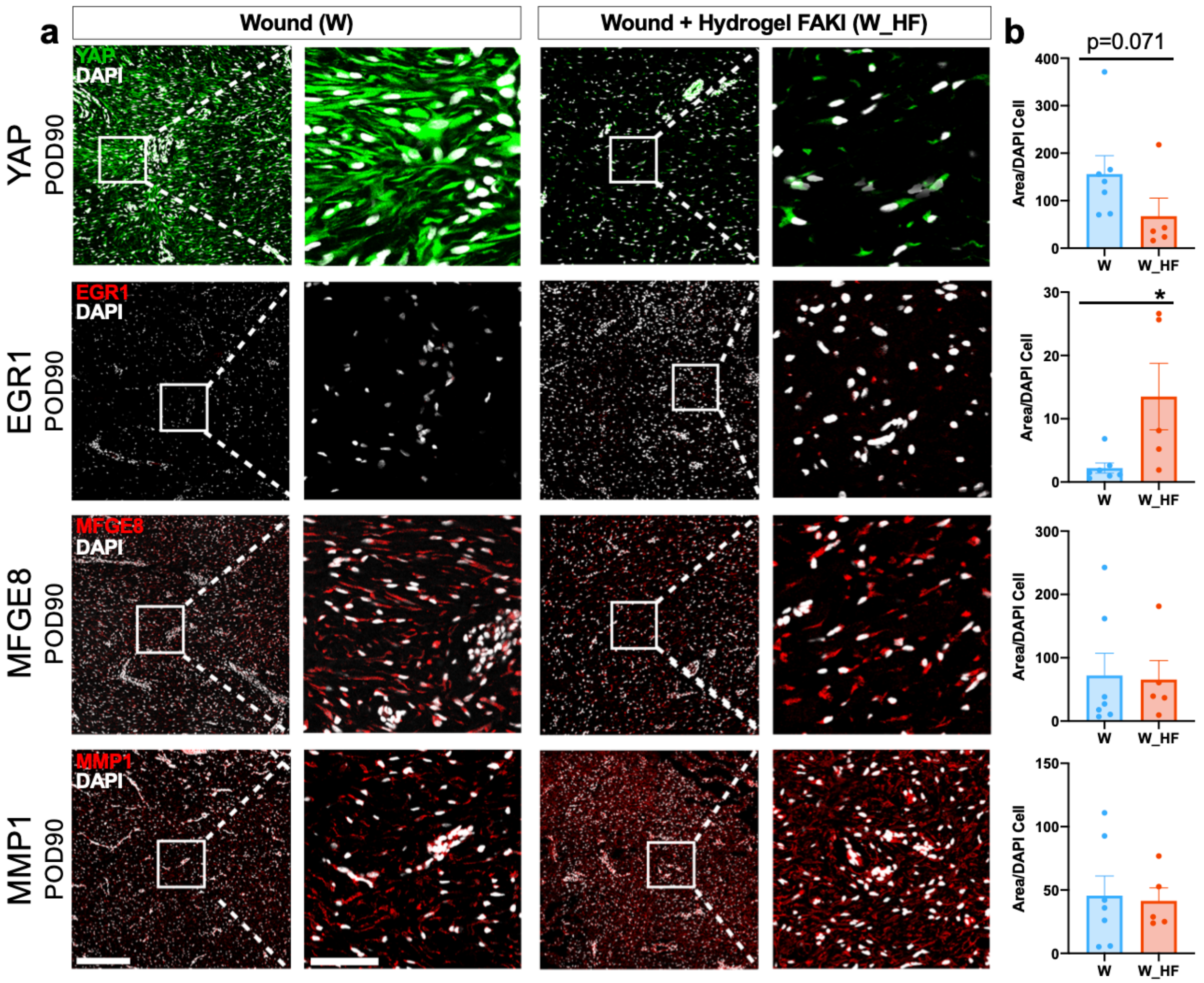

Supplementary Figure 9. Protein level confirmation of human scRNA-seq observations in large animal comparator at POD90. Protein level confirmation performed using immunofluorescence staining of wounded and untreated (W, left, n=7 independent wounds) vs. wounded and treated (W_HF, right, n=5 independent wounds) porcine dermis tissue sections at POD90 (supplement to Fig. 6a,b). **(a)** Staining and **(b)** quantification for YAP (the protein translated from *YAP1*), EGR1 (**p=0.0148*), MFGE8, and MMP1. *Scale bar: 200 μm. Magnified Image Scale Bar: 50 μm. Statistical comparisons were made using unpaired two-tailed t-tests. Each data point represents an independent wound. All data represent mean ± SEM. Representative images are shown from similar images across all experiments.*

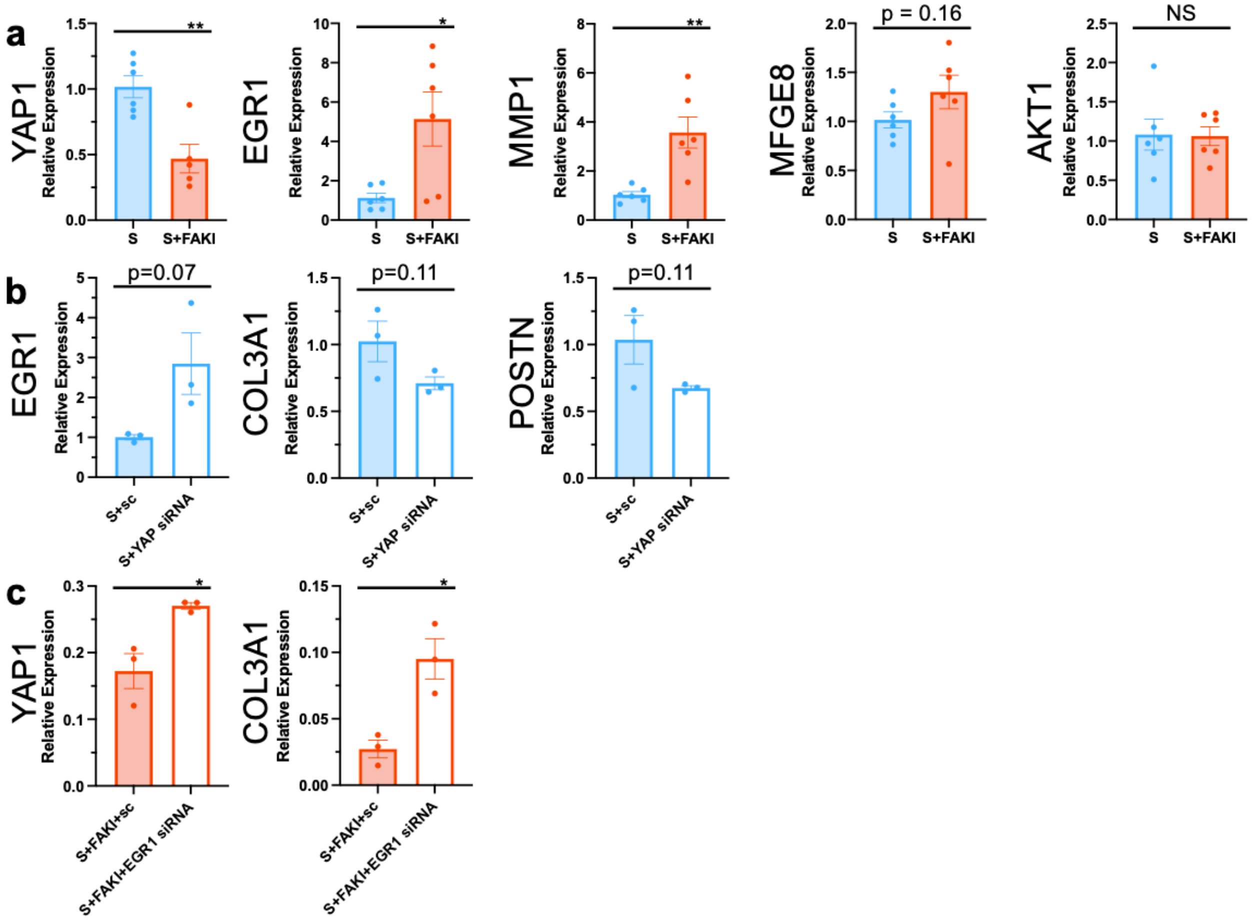

Supplementary Figure 10. **(a)** qPCR of human fibroblasts from repeated experiments utilizing our collagen scaffold system for YAP1 (n=5, **p=0.0028*), EGR1 (n=6, **p=0.0166*), MMP1 (n=6, ***p=0.0028*), MFGE8 (n=6), and AKT1 (n=6). **(b)** qPCR of human fibroblasts from YAP1 siRNA experiments utilizing our collagen scaffold system for EGR1 (n=6), COL3A1 (n=3), and POSTN (n=3). **(c)** qPCR of human fibroblasts from EGR1 siRNA experiments for YAP1 (n=3, **p=0.0214*) and COL3A1 (n=3, **p=0.0149*). S = strain (blue); S+FAKI = Strain + FAKI (red); sc = scrambled (control) siRNA (white bars). *Statistical comparisons were made using unpaired two-tailed t-tests. Each data point represents an independent collagen scaffold. All data represent mean ± SEM.*

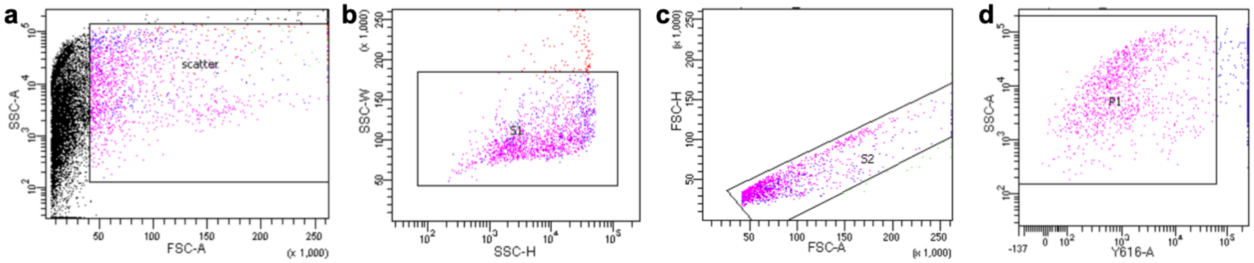

Supplementary Figure 11. Gating strategy used for cell sorting of fibroblasts from collagen scaffold debris before scRNA-seq. Cells were sorted from leftover collagen scaffold debris after enzymatic digestion along forward scatter (FSC) and side scatter (SSC) across both cell area and cell length. Cells were sorted from **(a)** to **(b)** to **(c)** to **(d)**. In **(d)**, cells were stained with propidium iodide and only live cells (unstained) were chosen. These cells were then submitted for scRNA-seq.

Gene Name	TaqMan Gene Expression Assay ID
GAPDH	Hs02786624_g1
MMP1	Hs00899658_m1
EGR1	Hs00152928_m1
YAP1	Hs00217433_m1
AKT1	Hs00178289_m1
MFGE8	Hs00983890_m1
COL3A1	Hs00943809_m1
POSTN	Hs01566750_m1

Supplementary Table 1. Complete list of all qPCR primers used.

Supplementary References:

1 Infante, J. R. *et al.* Safety, pharmacokinetic, and pharmacodynamic phase I dose-escalation trial of PF-00562271, an inhibitor of focal adhesion kinase, in advanced solid tumors. *Journal of clinical oncology : official journal of the American Society of Clinical Oncology* **30**, 1527-1533, doi:10.1200/jco.2011.38.9346 (2012).

2 Fomovsky, G. M. & Holmes, J. W. Evolution of scar structure, mechanics, and ventricular function after myocardial infarction in the rat. *American journal of physiology. Heart and circulatory physiology* **298**, H221-228, doi:10.1152/ajpheart.00495.2009 (2010).

3 Chen, K. *et al.* Role of boundary conditions in determining cell alignment in response to stretch. *Proceedings of the National Academy of Sciences of the United States of America* **115**, 986-991, doi:10.1073/pnas.1715059115 (2018).

4 Bredfeldt, J. S. *et al.* Computational segmentation of collagen fibers from second-harmonic generation images of breast cancer. *J Biomed Opt* **19**, 16007-16007, doi:10.1117/1.JBO.19.1.016007 (2014).

5 Liu, Y., Keikhosravi, A., Mehta, G. S., Drifka, C. R. & Eliceiri, K. W. Methods for Quantifying Fibrillar Collagen Alignment. *Methods Mol Biol* **1627**, 429-451, doi:10.1007/978-1-4939-7113-8_28 (2017).
