# Supplementary material for: Disrupting biological sensors of force promotes tissue regeneration in large organisms
Source: Nat Commun. 2021 Sep 6;12:5256. doi: 10.1038/s41467-021-25410-z (PMC8421385; doi:10.1038/s41467-021-25410-z)
Supplement: Supplementary file 2 — Reporting Summary [file 41467_2021_25410_MOESM2_ESM.pdf]

nature research

Corresponding author(s): Geoffrey C Gurtner

Last updated by author(s): Aug 2, 2021

Reporting Summary

Nature Research wishes to improve the reproducibility of the work that we publish. This form provides structure for consistency and transparency in reporting. For further information on Nature Research policies, see our Editorial Policies and the Editorial Policy Checklist.

Statistics

For all statistical analyses, confirm that the following items are present in the figure legend, table legend, main text, or Methods section.

n/a	Confirmed	
☐	☒	The exact sample size (*n*) for each experimental group/condition, given as a discrete number and unit of measurement
☐	☒	A statement on whether measurements were taken from distinct samples or whether the same sample was measured repeatedly
☐	☒	The statistical test(s) used AND whether they are one- or two-sided<br>*Only common tests should be described solely by name; describe more complex techniques in the Methods section.*
☒	☐	A description of all covariates tested
☐	☒	A description of any assumptions or corrections, such as tests of normality and adjustment for multiple comparisons
☐	☒	A full description of the statistical parameters including central tendency (e.g. means) or other basic estimates (e.g. regression coefficient) AND variation (e.g. standard deviation) or associated estimates of uncertainty (e.g. confidence intervals)
☐	☒	For null hypothesis testing, the test statistic (e.g. F, t, r) with confidence intervals, effect sizes, degrees of freedom and P value noted<br>*Give P values as exact values whenever suitable.*
☒	☐	For Bayesian analysis, information on the choice of priors and Markov chain Monte Carlo settings
☒	☐	For hierarchical and complex designs, identification of the appropriate level for tests and full reporting of outcomes
☒	☐	Estimates of effect sizes (e.g. Cohen's d, Pearson's r), indicating how they were calculated

Our web collection on statistics for biologists contains articles on many of the points above.

Software and code

Policy information about availability of computer code

Data collection	Cutometer data was captured using Cutometer Dual MPA 580 (v2020).
Data analysis	R packages Seurat (version 3.1.1), Monocle 3 (v0.2.1.2), scVelo (v0.2.3), GeneTrail (v3.0), SingleR (v3.11), CytoTRACE (code from https://cytotrace.stanford.edu) were used for single cell analyses. MatFiber code was previously developed and may be found on GitHub at https://github.com/cardiacbiomechanicsgroup/MatFiber.

For manuscripts utilizing custom algorithms or software that are central to the research but not yet described in published literature, software must be made available to editors and reviewers. We strongly encourage code deposition in a community repository (e.g. GitHub). See the Nature Research guidelines for submitting code & software for further information.

Data

Policy information about availability of data

All manuscripts must include a data availability statement. This statement should provide the following information, where applicable:

- Accession codes, unique identifiers, or web links for publicly available datasets
- A list of figures that have associated raw data
- A description of any restrictions on data availability

The authors declare that the source data supporting the findings of this study are provided with the manuscript and supplementary information files. The scRNA-seq data discussed in this publication have been deposited in NCBI's Gene Expression Omnibus and are accessible through GEO Series accession number GSE167339 (https://www.ncbi.nlm.nih.gov/geo/query/acc.cgi?acc=GSE167339). Automated cell-level annotations were ascribed using the SingleR toolkit (version 3.11) against the ENCODE blue database [reference in manuscript]. MatFiber code may be found on GitHub at https://github.com/cardiacbiomechanicsgroup/MatFiber. All other relevant data are available from the corresponding author on reasonable request.

Field-specific reporting

Please select the one below that is the best fit for your research. If you are not sure, read the appropriate sections before making your selection.

- [x] Life sciences
- [ ] Behavioural & social sciences
- [ ] Ecological, evolutionary & environmental sciences

For a reference copy of the document with all sections, see nature.com/documents/nr-reporting-summary-flat.pdf

Life sciences study design

All studies must disclose on these points even when the disclosure is negative.

Sample size

The wounds were randomly assigned to receive either FAKI hydrogel (W_HF), blank hydrogel ('placebo', W_H), or no hydrogel (wounded control, W) (n=6-9 wounds per condition). We could only perform experiments on 1 to 3 pigs concurrently. Additional experiments were performed as necessary to detect the effect of treatment across a range of variables.

We performed collagen scaffold experiments based on the availability of human clinical samples, which corresponded to the frequency of plastic surgery cases. Additional experiments were performed as necessary to detect the effect of treatment across a range of variables.

Data exclusions

No data were excluded.

Replication

To verify the reproducibility of our findings, we performed each major experiment multiple times in order to generate biological replicates. For pig studies, several pigs were used, with different treatment conditions distributed equally across each pig dorsum to minimize any inter-animal effects. All attempts at replication were successful.

For human studies, human fibroblasts were isolated from several different patients to replicate and repeat all experiments, including scRNA-seq (n=3), immunofluorescent staining (n=4), qPCR (n=6), western blot (n=4), and siRNA (n=3). This ensured that all findings were translatable and reproducible across different human samples. All attempts at replication were successful.

Randomization

Up to eight wounds, approximately 5cm x 5cm in size, were created on each lateral flank. The wounds were randomly assigned to receive either FAKI hydrogel (W_HF), blank hydrogel ('placebo', W_H), or no hydrogel (wounded control, W).

For each human patient sample experiment, 3 collagen scaffolds were seeded with the patient fibroblasts. These 3 collagen scaffolds would be randomly assigned to either No Strain, Strain, or Strain+FAKI groups.

Blinding

Days to wound closure were determined for each wound based on blinded assessment of gross photography. Quantification of scar metrics was performed using a Visual Analog Scale (VAS) for 5 components (vascularity, pigmentation, observer comfort, acceptability, and contour) by a panel of four blinded scar experts. Cutometer measurements were made blinded to wound assignment. Counting of hair follicles and glands were made blinded to the treatment allocation. Quantification of collagen staining as well as all immunofluorescent staining was made in an unbiased manner using MATLAB image processing to quantify the amount of stain in each sample. The same threshold was used for all images.

Picrosirius red staining quantification was made in an unbiased, quantitative manner using several previously published computer algorithms. The same threshold was used for all images.

qPCR was performed and quantified by a core facility (SFGF) blinded to the treatment allocation. Western blot quantification was made blinded to the lane allocation.

For scRNA-seq, cells were processed by a core facility (SFGF) blinded to treatment allocation. For analysis, data were normalized and processed according to the Seurat package in an unbiased manner.

Reporting for specific materials, systems and methods

We require information from authors about some types of materials, experimental systems and methods used in many studies. Here, indicate whether each material, system or method listed is relevant to your study. If you are not sure if a list item applies to your research, read the appropriate section before selecting a response.

Materials & experimental systems

n/a	Involved in the study
☐	☒ Antibodies
☒	☐ Eukaryotic cell lines
☒	☐ Palaeontology and archaeology
☐	☒ Animals and other organisms
☐	☒ Human research participants
☒	☐ Clinical data
☒	☐ Dual use research of concern

Methods

n/a	Involved in the study
☒	☐ ChIP-seq
☒	☐ Flow cytometry
☒	☐ MRI-based neuroimaging

Antibodies

Antibodies used

Immunofluorescent staining was performed using primary antibodies targeting EGR1 (1:100 dilution; ThermoFisher, PA5-83115), MFGE8 (1:100 dilution; ThermoFisher, PA5-82036), MMP1 (1:100 dilution; Abcam, ab52631), α-smooth muscle actin (1:200 dilution; Abcam, ab5694), YAP (1:100 dilution; CellSignaling, 14074S), and Perilipin-1 (1:100 dilution; Abcam, ab172907).

Immunoblotting analysis was performed using primary rabbit antibodies of YAP (1:200 dilution; Cell Signaling, 4912), EGR1 (1:500 dilution; Cell Signaling, 4153), MFGE8 (1:500 dilution; Sigma Aldrich, SAB1408603), MMP1 (1:1000 dilution; abcam, ab38929), and alpha-Tubulin (1:1000 dilution; Cell Signaling, 2125). A horseradish peroxidase-conjugated secondary anti-rabbit was used (1:2000 dilution; Cell Signaling, 7074).

Validation

All antibodies used were validated by the suppliers they were acquired from and were used at the optimized conditions according to the manufacturer's recommendations. Additional validation information and relevant publications are available on the manufacturers' websites.

Animals and other organisms

Policy information about studies involving animals; ARRIVE guidelines recommended for reporting animal research

Laboratory animals

Seven female red Duroc pigs, 6-8 weeks old and weighing approximately 16-20 kg at the time of surgery, were purchased from Pork Power Farms (Turlock, CA).

Wild animals

The study did not involve wild animals.

Field-collected samples

The study did not involve samples collected from the field.

Ethics oversight

All animal work was conducted in accordance with the Administrative Panel on Laboratory Animal Care protocols (APLAC# 31530 and 32962) approved by Stanford University.

Note that full information on the approval of the study protocol must also be provided in the manuscript.

Human research participants

Policy information about studies involving human research participants

Population characteristics

N/A.

Demographic data was not collected.

Recruitment

N/A.

We have complied with all relevant ethical regulations for work with human tissue, and all human skin was collected with ethical approval under the IRB #54225 from Stanford University. Skin tissue was collected from patients undergoing procedures which normally involve tissue removal (including plastic surgery operations). This skin would otherwise be discarded as medical waste. Thus, there was no additional risk to patients and recruitment was not needed.

The samples were collected by co-authors (MRB and JB) who were not the primary investigators of this research study. These samples were collected in an unbiased manner, based solely on clinical availability.

Ethics oversight

Human skin samples were obtained under the approved IRB (#54225) by Stanford University.

Note that full information on the approval of the study protocol must also be provided in the manuscript.
