# Supplementary figures and images for: Disrupting biological sensors of force promotes tissue regeneration in large organisms
Source: Nat Commun. 2021 Sep 6;12:5256. doi: 10.1038/s41467-021-25410-z (PMC8421385; doi:10.1038/s41467-021-25410-z)

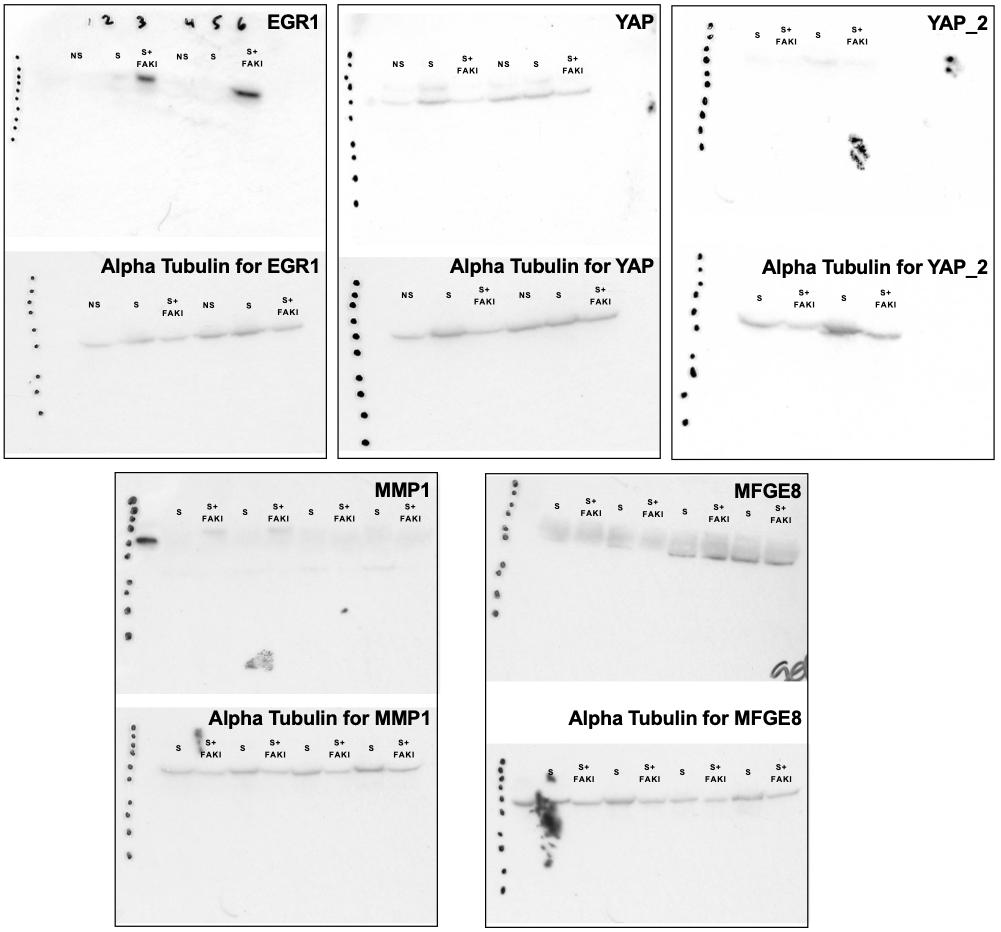

Supplement: Supplementary file 3 — Source Data [file 41467_2021_25410_MOESM3_ESM.zip › Source Data/WesternBlot_Raws_withLadders.tiff]
